# Auditory cues reveal intended movement information in middle frontal gyrus neuronal ensemble activity of a person with tetraplegia

**DOI:** 10.1038/s41598-020-77616-8

**Published:** 2021-01-11

**Authors:** Tommy Hosman, Jacqueline B. Hynes, Jad Saab, Kaitlin G. Wilcoxen, Bradley R. Buchbinder, Nicholas Schmansky, Sydney S. Cash, Emad N. Eskandar, John D. Simeral, Brian Franco, Jessica Kelemen, Carlos E. Vargas-Irwin, Leigh R. Hochberg

**Affiliations:** 1 School of Engineering, Brown University, Providence, RI USA; 2 Robert J. and Nancy D. Carney Institute for Brain Science, Brown University, Providence, RI USA; 3 Department of Neuroscience, Brown University, Providence, RI USA; 4 Neuroscience Graduate Program, Brown University, Providence, RI USA; 5 Center for Neurorestoration and Neurotechnology, Rehabilitation Research and Development Service, Department of Veterans Affairs Medical Center, Providence, RI USA; 6 Department of Radiology, Massachusetts General Hospital, Boston, MA USA; 7 Athinoula A. Martinos Center for Biomedical Imaging, Massachusetts General Hospital, Boston, MA USA; 8 Center for Neurotechnology and Neurorecovery, Department of Neurology, Massachusetts General Hospital, Boston, MA USA; 9 Department of Neurology, Harvard Medical School, Boston, MA USA; 10 Department of Neurosurgery, Massachusetts General Hospital, Boston, MA USA; 11 Present Address: Department of Neurosurgery, Albert Einstein College of Medicine, Montefiore Medical Center, New York, NY USA; 12 Present Address: NeuroPace, Inc., Mountain View, CA USA

**Keywords:** Brain-machine interface, Premotor cortex, Translational research

## Abstract

Intracortical brain-computer interfaces (iBCIs) allow people with paralysis to directly control assistive devices using neural activity associated with the intent to move. Realizing the full potential of iBCIs critically depends on continued progress in understanding how different cortical areas contribute to movement control. Here we present the first comparison between neuronal ensemble recordings from the left middle frontal gyrus (MFG) and precentral gyrus (PCG) of a person with tetraplegia using an iBCI. As expected, PCG was more engaged in selecting and generating intended movements than in earlier perceptual stages of action planning. By contrast, MFG displayed movement-related information during the sensorimotor processing steps preceding the appearance of the action plan in PCG, but only when the actions were instructed using auditory cues. These results describe a previously unreported function for neurons in the human left MFG in auditory processing contributing to motor control.

## Introduction

Intracortical brain-computer interfaces (iBCIs) record signals directly from the brain and harness those signals to control external devices, such as computer cursors or robotic arms^1–12^. This technology can also be used to directly reanimate paralyzed muscles using functional electrical stimulation^13–17^. Using these techniques, iBCI systems have the potential to restore communication and mobility for people with paralysis resulting from syndromes of abrupt onset (e.g., spinal cord injury, stroke) or more progressive, neurodegenerative disorders (e.g. amyotrophic lateral sclerosis). Selecting area(s) of the brain to record from is a critical decision in deploying an iBCI. Voluntary movement generation engages a wide constellation of cortical areas^18,19^. Realizing the full potential of iBCI technology will benefit from acquiring a deeper understanding of how movement-related information is encoded across these broad cortical networks^20,21^. Most iBCI research in humans has focused on recording neural activity from precentral gyrus (PCG)^14,15,22^, the single anatomical location with the greatest number of direct projections to the spinal cord in primates^23^. More recently, neural activity from posterior parietal cortex (PPC), an area linked to sensorimotor integration and formulating movement goals, was also used with an iBCI to control a robot arm^24,25^.

Cortical areas engaged in early sensorimotor transformations related to action planning^26^ present another promising source of signals for iBCI systems. Recent work in non-human primates (NHPs) has highlighted the potential of this strategy by comparing signals obtained in primary motor cortex (M1) with premotor cortex (PMC). In non-human primates, PMC contributes about 70% of the frontal cortico-cortical connections to the upper limb area of M1^19^, and approximately one-third of the corticospinal connections to the upper cervical spinal cord^23^, suggesting that PMC has an important role in initiating and controlling movements of the arm and hand. PMC is also involved in higher order features of motor control, such as dynamically updating movement plans^27^ and encoding the relative position of the eyes, body, and endpoint of the action to facilitate coordination^28^. Individual neurons in NHP dorsal PMC (PMd) have been shown to respond during instructed delay periods before the execution of a movement^29–31^, providing an early signal about the intended motion. This activity may reflect a variety of features of the upcoming movement, including its direction and amplitude^32^, speed^33,34^, and reaction time^35,36^. Furthermore, iBCI control in NHPs using signals from PMd resulted in substantial performance improvements^5^ as compared to previous studies using only neural signals from M1.

This study was designed to explore neuronal ensemble activity potentially related to movement planning in a cortical site anterior to the PCG. We recorded neural activity in a participant in the BrainGate2 pilot clinical trial using two Blackrock microelectrode arrays^37^. One array was placed in the hand/arm knob area of the PCG^38^, a site that has consistently provided signals related to the intention to move the contralateral arm and hand. The second array was implanted into the caudal middle frontal gyrus (MFG), in a location selected using an fMRI protocol designed to highlight cortical areas activated during movement planning.

The present work constitutes the first comparison between human PCG and MFG ensemble activity at the scale of individual neurons. Our results show that human MFG differs markedly from PCG, revealing activity that highlights the interpretation of auditory cues in the service of movement guidance. We also observed that similar numbers of MFG neurons were active during both intended hand movements and movements of the eyes alone, suggesting that MFG activity is less specific to motor effector than PCG activity.

## Results

**Figure 1:**
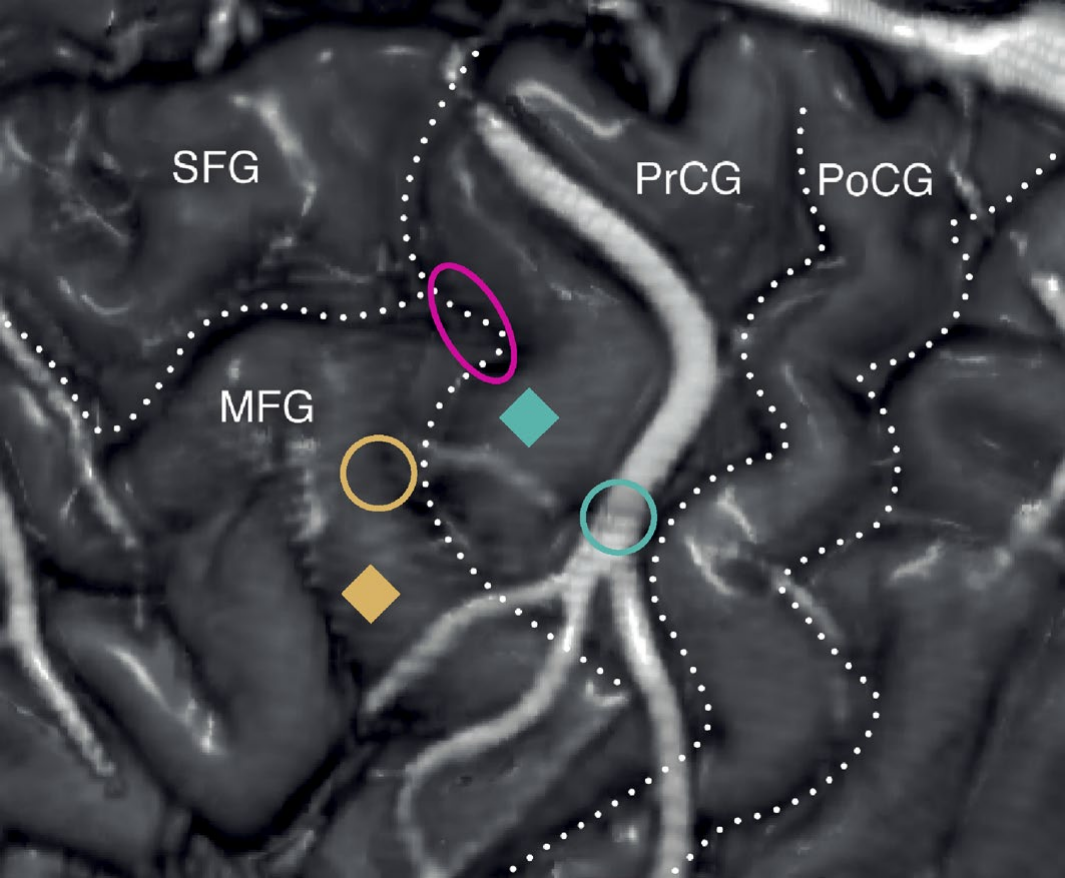
Anatomical location of implanted PCG and MFG microelectrode arrays in relation to different movement-related fMRI activation signals. Volume rendering of MRI images with intravenous contrast delineating surgically important cortical venous landmarks. Anatomical locations of surgically implanted PCG (*teal square*) and MFG (*yellow square*) microelectrode arrays shown in relation to three key fMRI activation foci associated with attempted reach-and-grasp movements (*teal circle*), movement planning (*yellow circle*), and eye movement (*magenta ellipse*). The three circular shapes circumscribe the maximal peak fMRI activation, as calculated using a voxel-based and surface-based fMRI analysis; see Supplementary Materials and Figs. S4–S7 for full details. Dotted lines highlight superior frontal, precentral, central, and postcentral sulci. SFG, Superior frontal gyrus; MFG, Middle frontal gyrus; PrCG, Precentral gyrus; PoCG, Postcentral Gyrus. After array implantation, electrophysiological data was collected during a separate set of closed-loop iBCI tasks. This figure was compiled using MRIcro v. 1.9.0 and labeled using Adobe Illustrator CC 2019.

We used structural and functional imaging to identify regions of maximal activation on the cortical surface associated with movement preparation, intended movements of the right hand, and eye movements in a participant designated as T10, a 34 year-old man with chronic tetraplegia, secondary to C4 AIS-A spinal cord injury (see Supplementary Materials and Fig. S4–S7 for full fMRI details). Microelectrode arrays were implanted as close as possible to the fMRI-based target locations associated with action planning (MFG site) and attempted reaching and grasping movements (PCG site) given the anatomical and vascular constraints, while avoiding the area most closely associated with eye movements (assumed to be a core component of the frontal eye field) (Fig. 1). We note that the transition from Brodmann’s area 4 (primary motor cortex) to area 6 (premotor cortex) is gradual in both NHPs and humans. For the purpose of this paper, we will conservatively describe the location of cortical recordings (i.e., the location of the cortical implants) anatomically instead of assigning the recorded activity to a specific cytoarchitectural area from which function is often inferred (see Supplementary Materials for further discussion). Following implantation, extracellular voltage at each electrode was sampled at 30 kHz during recording sessions. For simplicity, well-isolated extracellular single unit signals are referred to as ‘neurons’ throughout this report (see Materials for signal processing details).

Prior to the work described here, we performed a preliminary examination of activity in PCG and MFG during a standard 2D iBCI task where T10 controlled a cursor using neural activity associated with intended right-hand movements. Although individual neurons in PCG presented clear modulation according to the participant’s intended movement direction, MFG neurons displayed a surprising lack of direction-related modulation during active iBCI control (Supplementary Fig. S1). This observation was contrary to expectations based on a previous acute single electrode study in humans^39^ and large-scale recording studies in NHPs^28–32,40^. In order to better understand the response properties of the MFG recording site, the next phase of our study focused on comparing neural activity during planning and execution of eye movements compared to intended hand movements (see Supplementary Materials and Fig. S2). This “Eye-Hand” task used simultaneous auditory and visual cues in order to make the targets more salient (Supplementary Fig. S2). Surprisingly, the addition of auditory cues revealed movement-related information in MFG, in stark contrast to previous results using only visual cues. Additionally, MFG activity elicited by audio-visual cues was equally informative about eye and intended hand movement direction, in sharp contrast to PCG, where intended hand movement was clearly dominant (Supplementary Fig. S3). Based on these results, we designed a Multi-modal task aimed at characterizing the neural responses in MFG and PCG elicited by auditory and visual cues presented independently or in combination.

**Figure 2:**
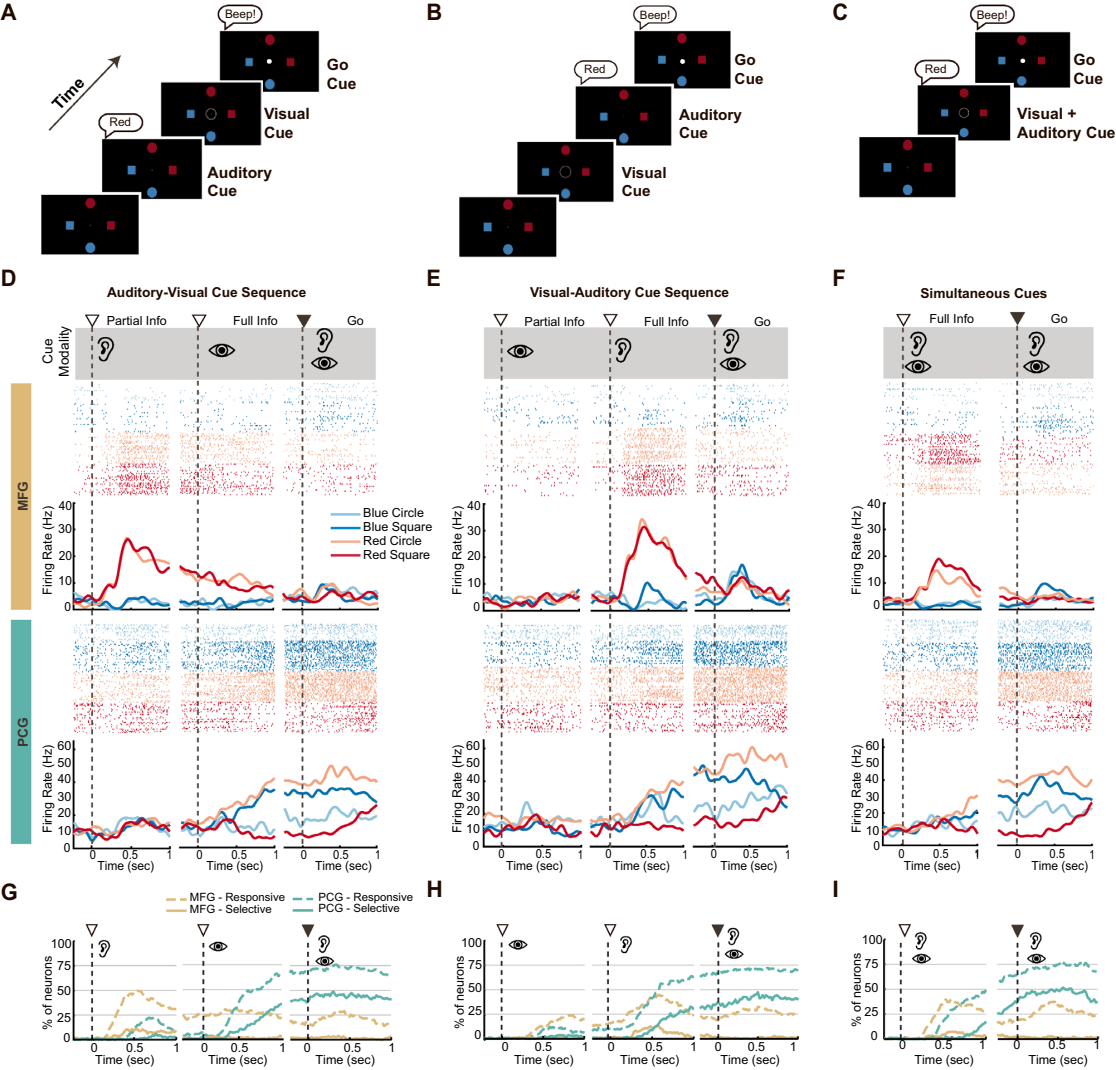
Single neuron responses to auditory and visual cues during three different variations of a Multi-modal center-out iBCI task. (**A**–**C**) Task sequences for three variants of a Multi-modal center-out task consisting of different cue presentation orders: auditory-visual (AV) task sequence (**A**), visual-auditory (VA) task sequence (**B**), and the simultaneous cue (A + V) presentation sequence (**C**). (**D**–**F**) Example raster-histograms for a single MFG neuron (*top row, yellow banner*) and PCG neuron (*bottom row, teal banner*) during the AV task sequence (**D**), the VA task sequence (**E**), and the A + V task sequence (**F**). Averaged firing rate traces for each target (smoothed with a 200 ms Gaussian kernel) are presented under each raster plot. White triangles (∇) represent the instructional cue onsets; black triangles represent the go cue onset; Ear symbol represents an auditory cue; eye symbol represents a visual cue (trial day 356, MFG channel 17, unit 1, PCG channel 12, unit 3). (**G**–**I**) Percentages of MFG (*yellow line*s) and PCG neurons (*teal lines*) with responsive (*broken line*) and selective (*solid line*) firing patterns during the AV (**G**), the VA (**H**), and the A + V (**I**) task sequence. Neurons were classified as responsive if they displayed firing rates significantly different from baseline during task performance (significance level = 0.01; Kruskal–Wallis (KW) test), and selective if firing rates differed significantly across targets. This figure was compiled using MATLAB 2017b, MathWorks software and labeled using Adobe Illustrator CC 2018.

In order to explore the role of MFG in auditory/visual cue processing, we designed a 2D radial “center-out” target selection task wherein the participant had to combine visual and auditory information in order to determine which target to aim for (Fig. 2), the Multi-modal Cues task (see iBCI Multi-modal Cues task in Materials and Methods). In order to select targets, T10 moved a cursor using closed-loop neural control implemented using a Kalman filter trained on intended right-hand movements (see Supplementary Materials for calibration details). The four possible targets represented all combinations of two shapes (circle or square) and two colors (red or blue). The position of the “correct” target on a given trial was indicated to T10 using a partially informative visual cue (a circle or square) and a partially informative lexical auditory cue (the word “red” or “blue”) that, when combined, unambiguously identified a single target. The auditory and visual instructional cues were presented during the instructed delay period in either a sequential manner (AV, audio cue first trials, Fig. 2A; VA, visual cue first trials, Fig. 2B); or a simultaneous manner (A + V, simultaneous audio and visual trials, Fig. 2C). The different cue presentation orders (AV, VA, and A + V) allowed the determination of whether MFG neuron selectivity was tied to the presentation of an action-relevant auditory cue or the requirement to integrate an auditory and visual cue during action planning. For all trial types, the go cue had both an auditory ‘beep’ and visual dimming of the center circle and brightening of the cursor. Importantly, these go cues did not provide target information.

### MFG neurons respond selectively to auditory cues during movement planning

We recorded extracellular neurophysiological signals from the PCG and MFG arrays on post-implant trial days 356, 357, and 362 while participant T10 performed the three different variants of the Multi-modal iBCI center-out task. We then characterized the informational content of the PCG and MFG neural signals at the individual-neuron and ensemble levels.

Individual neurons were characterized as being either ‘responsive’ or ‘selective’, where neurons that displayed significantly different firing rates during either the delay or go epochs compared to baseline were labeled as ‘responsive’ (Kruskal–Wallis (KW), *p* < 0.01). Neurons that exhibited distinct changes in firing rate for at least one of the possible targets (KW, *p* < 0.01) were considered ‘selective’ (Fig. 2D–F example MFG and PCG neuron, both with a responsive and selective categorization). Firing rates were binned using a sliding 300 ms time window, shifted in 20 ms increments.

A greater proportion of MFG neurons were active in periods following auditory cues as compared to visual cues (Fig. 2G,H, see “Partial Info” cue phase). During the auditory phase of the AV trials (Fig. 2G, dashed yellow line during “Partial Info”), ~ 46% of the neurons were responsive compared to ~ 22% for the visual phase of the VA trials (Fig. 2H, dashed yellow line during “Partial Info”). Likewise, up to 22% of neurons were determined to be selective following the first auditory cue (Fig. 2G, solid yellow line), compared to only 7% when the visual cue was provided first (Fig. 2H, solid yellow line). When the audio and visual cues were presented simultaneously during the planning phase of the A + V trials (Fig. 2I), the firing response patterns of the MFG neurons closely resembled those observed during the AV trials, when only the auditory cue was presented (Fig. 2G). Selective activity was almost completely absent in MFG during the go phase of the Multi-modal task, even though a third of the neurons were active above baseline levels, i.e., were responsive. In contrast, we found that PCG activity patterns displayed little variation across AV, VA, and A + V conditions (Fig. 2G–I, teal lines). PCG neurons were minimally responsive or selective until both the auditory and visual cues had been presented (i.e., after the target location could be unambiguously identified from the cues): following the second cue—either auditory or visual—the number of responsive and selective neurons increased in a monotonic fashion. In the go phase, PCG was robustly engaged: 72–81% of neurons were classified as responsive, and 58–64% as action selective. Overall, responsive and selective neurons in MFG had shorter response latencies than in PCG (with MFG leading by ~ 200 ms, on average). A detailed analysis of single neuron response latency is presented in a separate section below.

**Figure 3:**
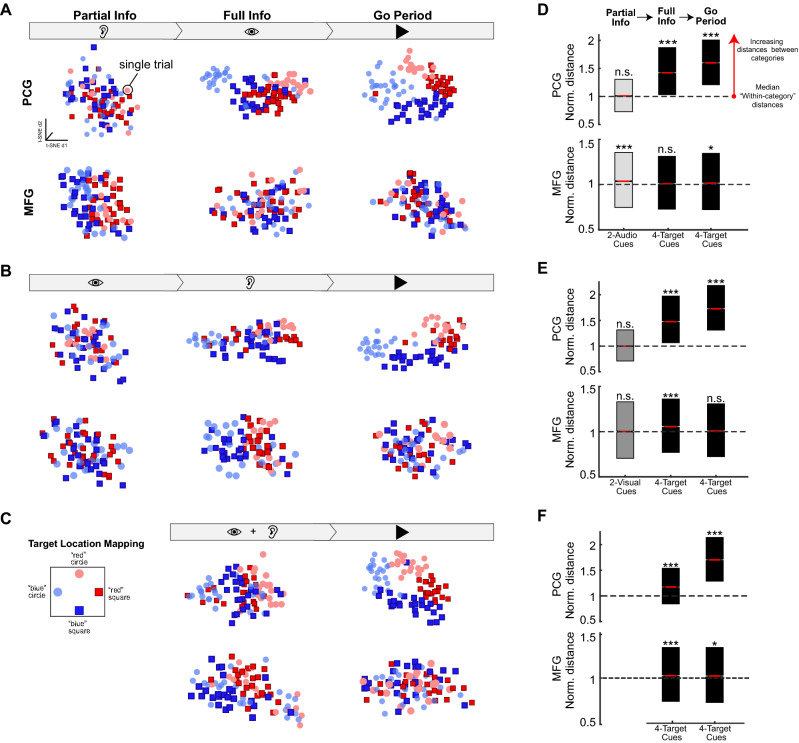
Spike train similarity analysis reveals that MFG displays distinct, recurring single trial activity patterns in response to partially informative auditory cues, while movement-specific activity emerges in PCG only after targets have been unambiguously specified. Spike train similarity space (SSIMS) representation of PCG (*top row*) and MFG (*bottom row*) ensemble spiking responses (one session, day 356) during the presentation of the target-related auditory cues (*ear symbol*), target-related visual cues (*eye symbol*), and the audio-visual go cue (*black triangle*) in the AV task (**A**), the VA task (**B**), and the A + V task sequences (**C**). Each data point represents the ensemble spiking pattern on a single trial and the distance between points corresponds to the degree of dissimilarity between the ensemble spiking pattern on those trials. The shape and color of each data point indicates which visual cue (circle or square) and auditory cue (“red” or “blue) were presented on a given trial (schematic of target locations used for this recording session are shown in **C**). (**D-F**) Boxplots show the normalized average distances between different sets of data points in the PCG (*top row*) and MFG (*bottom row*) SSIMS representations during different phases of the AV (**D**), VA (**E**), and A + V (**F**) task sequences (pooled across sessions).The distributions of “Between-category” distances were normalized against the median (*black dashed line*) of the distances between points representing a common category of trials (“Within-category” distances). A KW test was used to assess the statistical significance of any differences among the normalized Within- and Between-category distributions, pooled across sessions (*, p < 0.05; ***, p < 0.001; n.s, not significant, *p* >  = 0.05; see Table 1 for all *p*-values). Significant distance values above 1 indicate that the average distance between trials from different clusters is larger than the distance between trials within a cluster, i.e., non overlapping medians. Lower and upper edges of box-plots indicate the 25th and 75th percentiles of the distributions, respectively. Red markers indicate medians. This figure was compiled using MATLAB 2019, MathWorks software and labeled using Adobe Illustrator CC 2019.

The next goal was to characterize the nature of the information encoded by the MFG and PCG ensembles across different phases of the AV, VA, and A + V Multi-Modal task sequences (Fig. 3). To this end, we used spike train similarity space (SSIMS) analysis^41^ to quantify differences in the temporal structure of the single-trial ensemble spiking patterns for different phases of the Multi-modal tasks. This allowed us to quantify the discriminability of the relevant task variables (i.e., auditory cues, visual cues, target location) based on the collective spiking patterns generated by the PCG and MFG ensembles on a trial-by-trial basis. Briefly, SSIMS analysis begins by using spike train edit-distances^42^ to quantify the similarity between ensemble spiking patterns on individual trials. A dimensionality reduction method (in this case, t-SNE^43^) is then used to map the (high-dimensional) pairwise similarity measures into a low-dimensional space that preserves local neighborhood structure, highlighting the relationship between single-trial ensemble responses (See Methods for SSIMS analysis details). Points in a SSIMS plot represent ensemble-wide firing patterns, and the distance between the points conveys the degree of similarity between them (with similar patterns close together, and increasingly dissimilar patterns further apart).

Using this relational encoding analysis framework, we generated separate MFG and PCG ensemble SSIMS representations for each phase of the AV, VA, and A + V task sequences (Fig. 3A–C) and computed the pairwise distances distributions for different trial type categories to determine the overlap between the neural sub-spaces associated with the auditory cues, visual cues, and intended actions/targets (Fig. 3D–F; see Table 1 for KW *p*-values).

**Table 1:** KW test p-values for comparison of SSIMS distance distributions.

	Partial info		Full info		Go period	
	**PCG**	**MFG**	**PCG**	**MFG**	**PCG**	**MFG**
AV Task	0.055 n.s	2.15 × 10^–4^ ***	0.0000 ***^+^	0.128 n.s	0.0000 ***^+^	0.022 *
VA Task	0.252 n.s	0.274 n.s	0.0000 ***^+^	3.37 × 10^–22^ ***	0.0000 ***^+^	0.884 n.s
V + A Task			1.37 × 10^–54^ ***	2.46 × 10^–9^ ***	0.0000 ***^+^	0.047 *

n.s., not significant; *, *p* < 0.05; ***, *p* < 0.001; ***^+^, *p*-value < 1 x 10^-300^ (minimum floating point accuracy in Matlab).

SSIMS analysis revealed a clear structure across spiking patterns in PCG, resulting in clusters of trials associated with each of the four targets, which emerged after both cues were provided (and the target was unambiguously specified, Fig. 3D–F; see “Full Info” and “Go Period” in top row). This pattern was observed regardless of the task sequence (either AV, VA, and A + V, Fig. 3A–C; top row). For PCG, the relative position of the trial clusters in the “Go period” SSIMS representations matched the spatial relationship of the targets relative to one another (see Fig. 3C for map of target layout). Note that this pattern resulted solely from the intrinsic relationships between the spiking patterns, since SSIMS is an unsupervised dimensionality reduction algorithm.

By contrast, MFG ensemble firing patterns for individual trials did not form clusters associated with specific targets in the SSIMS representations. Instead, the intrinsic structure of the single-trial responses revealed consistent patterns associated with each of the auditory cues for every task phase when they were presented (Fig. 3A–C, columns with “ear” symbol). Note that for the AV condition, clustering according to auditory cues was present in MFG before any pattern was evident in PCG. In line with our previous findings from the single neuron analysis, these results suggested that MFG was engaged in processing information related to auditory cues, even before the target was fully specified.

**Figure 4:**
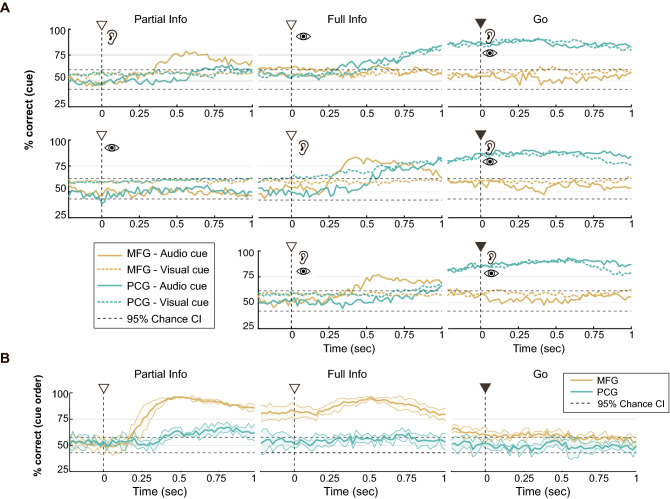
MFG is specifically engaged during auditory cue processing and not visual or multi-modal cue processing. (**A**) Classification accuracies of SVM models designed to distinguish trials on which the target was cued with different auditory cues (*solid lines*; red vs blue auditory cues) or different visual cues (*dashed lines*; square vs circle visual cues) using ensembles of MFG (*yellow*) and PCG (*teal*) neurons in the AV task sequence (*top row*), VA task sequence (*middle row*), and A + V task sequence (*bottom row*) in the Multi-modal Cues task. (**B**) Classification accuracies for discrimination between AV versus VA trials using MFG *(yellow)* and PCG (*teal*) ensemble activity in the Multi-modal Cues task. This figure was compiled using MATLAB 2019, MathWorks software and labeled using Adobe Illustrator CC 2019.

The next part of our analysis used predictive decoding models to more precisely compare the informational content of the PCG and MFG neuronal ensembles across the AV, VA, and A + V tasks (Fig. 4). We used a multiclass support vector machine (SVM) with five-fold cross-validation to predict targets for a given cued modality for each trial based on the activity of all neurons in each area within a sliding 300 ms window (Fig. 4A). We separately tested the prediction of the target color (red vs. blue auditory cues) and shape (square vs. circle visual cues) to determine the effect of cue modality on information processing across the full time-course of the tasks.

We found that PCG activity could be used to decode auditory cued targets and visually cued targets with approximately 90% accuracy in all three trial types (AV, VA, or A + V) during the full information and go phases (Fig. 4A; dashed and solid teal lines). By contrast, MFG ensembles only displayed classification accuracy above expected chance levels following the auditory cues (Fig. 4A; solid yellow lines). It was not possible to discriminate visually cued targets above chance levels based on MFG activity (Fig. 4A; dashed yellow lines).

Our observation that PCG displayed selective activity patterns only after both instruction cues were provided (i.e., once movement target was unambiguously specified) suggested that PCG activity was more closely tied to the neural processes underlying the selection of an appropriate motor/action plan than the earlier, perceptual stages of movement planning. To address this question, we performed a 2-way classification analysis on the AV and VA trials leading to the same intended movement in order to test the hypothesis that PCG would be insensitive to the cue presentation order, and only reflect the final intended motor output (Fig. 4B). Although we observed a small, significant peak in classification performance for PCG after the first cue, classification accuracy remained around the 95% confidence interval (CI) for most of the instructed delay period and the go period (Fig. 4B, teal lines). In contrast, classification of MFG's activity rose sharply approximately 200 ms following the first, partially informative cue and remained consistently above 75% accuracy throughout the instructed delay period before eventually dropping below chance during the go period.

Taken together, our finding from the single neuron and ensemble analyses suggest that information present in PCG during the later stages of the delay period is associated with neural processes that occur during action selection, while the information present in MFG is more closely tied to the presence of the action-relevant auditory cues.

### MFG neurons respond earlier than PCG neurons

**Figure 5:**
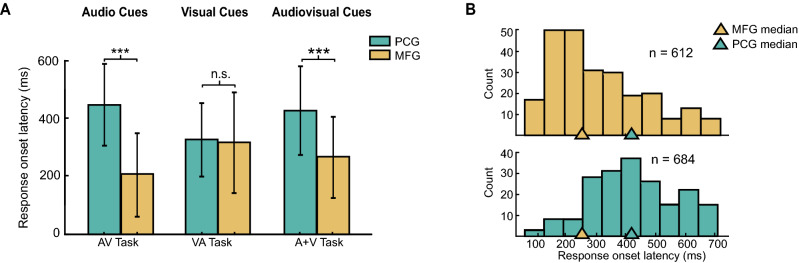
Average MFG neuron response onset precedes that of PCG. (**A**) Median response onset latencies for all PCG (*teal*) and MFG (*yellow*) single neurons following cue onset in the AV, VA, and A + V trials of the Multi-modal task. PCG bar plots represent an average of n = 20 (AV), 13.6 (VA), and 32.3 (A + V) latency values per session, while the MFG bar plots represent an average of n = 41.6 (AV), 20.6 (VA), 34.3 (A + V) latency values per session. Latencies were pooled across all three data collection sessions for each trial type. A KW test was used to assess statistical significance between each pair of MFG and PCG distributions (***, *p* < 0.001; n.s., p >  = 0.05). (**B**) Histograms of PCG (*teal*) and MFG (*yellow*) single neuron response onset latencies pooled across all three conditions referenced in (**A**), where n = number of neurons. Teal and yellow triangles represent the median response onset latencies PCG and MFG, respectively. This figure was compiled using MATLAB 2019, MathWorks software and labeled using Adobe Illustrator CC 2019.

We next compared the response onset latencies of PCG and MFG single neurons to the initial cues during the AV, VA, and A + V trials of the Multi-modal task to determine if MFG neurons are activated earlier than PCG neurons during action planning (Fig. 5).

MFG neurons responded substantially earlier than the PCG neurons during both the AV trials (median difference = 240 ms, *p* = 3.6 × 10^–17^; KW test) and A + V trials (median difference = 140 ms*, p* = 3.5 × 10^–8^; KW test), but had a similar response onset latency during the VA trials (median difference = 20 ms, *p* = 0.615; KW test) (Fig. 5A). When we compared the response onset latencies of a given ensemble across the AV trials (auditory cue) and VA trials (visual cue), we found that the MFG latencies were substantially faster during the AV trials (median difference = 120 ms; *p* = 3.5 × 10^–6^, KW test) but that the PCG response latencies were faster during the VA trials relative to the AV trials (median difference = 100 ms*, p* = 0.002, KW). These results indicate that the response onset latencies of both the MFG and PCG neurons are dependent upon cue modality.

An additional investigation into the neuronal source of the modality-dependent latency changes revealed that the PCG neurons that were active during *both* the AV and VA trials had similar onset latencies (median difference = 20 ms; *p* = 0.4966; KW test), while the PCG neurons that were *exclusively* active during the AV trials exhibited a delayed response onset when compared to latencies for the VA trials (median difference = 140 ms; s.d. difference = 10 ms; *p* = 5.4 × 10^–5^, KW test). In contrast, the MFG neurons that were active during *both* the AV and VA trials tended to respond earlier during the AV trials than the VA trials (median difference = 160 ms; s.d. difference = 32 ms; *p* = 2.9 × 10^–6^, KW test). In addition, the population of MFG neurons that were *exclusively* active during the AV trials exhibited a faster response onset latency than the latencies for VA trials (median difference = 120 ms, s.d. difference = 25 ms; *p* = 2.85 × 10^–4^, KW test).

In summary, the median response onset latency of the MFG neurons preceded that of PCG neurons by about 160 ms (s.d. = 146 ms) (Fig. 5B); however, these latency differences were modality dependent (auditory versus visual), where MFG neurons responded earlier in the presence of an auditory stimulus (~ 120 ms) and the PCG neurons exhibited a delayed response in the presence of an auditory stimulus (~ 100 ms).

### MFG and PCG neurons reveal emergent selectivity during presentation of task-relevant auditory stimuli

Results from the Multi-modal task suggested that MFG is selectively engaged during auditory cue processing; however, it remained unclear if this response was tied to the behavioral relevance of the auditory cues for action selection, i.e., action-relevant sounds, or if MFG neurons possess an inherent selectivity for certain categories of sound, e.g., lexical, action-related words, or neutral tones, independently of their relevance to action/movement planning. To address this question, we examined MFG and PCG neuron responses at the single-neuron and ensemble level during a Passive Listening paradigm in which T10 simply listened to a set of six auditory stimuli while visually fixating a cross at the center of the screen; no intended movement or other response was required.

**Figure 6:**
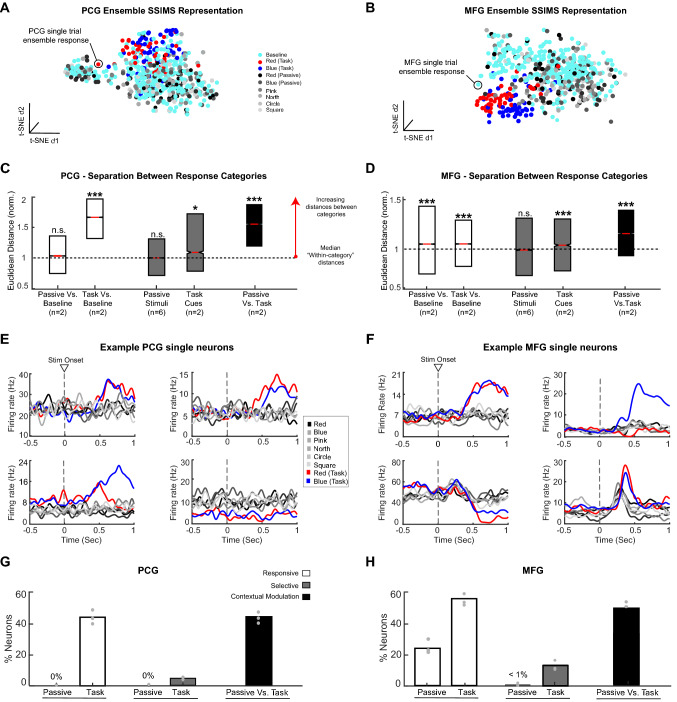
MFG displays non-selective responses to different sounds during passive listening, while PCG responds to sound only within the context of the behavioral tasks. (**A**,**B**) SSIMS representations of PCG (**A**) and MFG (**B**) ensemble responses (day 356) during the presentation of non-instructional auditory stimuli under a Passive Listening paradigm (*gray points*), the instructional auditory cues during the Multi-modal task (*red and dark blue points*), and a baseline period (*cyan point*s). The proximity of the points reflects the similarity of the ensemble responses on those trials. (**C,D**) Boxplots show the degree of separation (median Euclidean distance) between different categories of trials (i.e., points) in the PCG (**C**) and MFG (**D**) SSIMS plots, for the following categories: between the baseline responses and non-instructional auditory stimuli (*Passive; white box*); the baseline responses and instructional auditory cues (*Task; white box*); among the six different sounds during the Passive Listening context (*gray box*); among the two different sounds during the Multi-modal task (*gray box*); and responses to sounds across the two different listening contexts (*black box*; context-dependent encoding of sounds). A KW test was used to assess significance among the distribution of “Between-category” and “within-category” distances (***, *p* < 0.001; *, *p* < 0.01; n.s. = not significant, *p* >  = 0.05). See Methods and Fig. 2. for additional details. (**E,F**) Trial-averaged time-varying firing rates of four example PCG (**E**) and MFG (**F**) neurons to the non-instructional auditory stimuli (*gray solid lines*) and instructional auditory cues (*red/blue lines*). Dashed lines indicate stimulus onset timing. Note, example PCG neurons (**E**) only responded to the instructional auditory cues and not to any of those same auditory stimuli when presented in a non-instructional context (Passive Listening), while some MFG neurons (**F,** *bottom row*) were responsive to the non-instructional auditory stimuli (Passive Listening) but did not discriminate among the different auditory stimuli (Passive Listening). (**G**,**H**) Proportions of PCG and MFG neurons that were responsive (*white bars*) or selective (*gray bars*) for the instructional auditory cues (Passive) or non-instructional auditory stimuli (Task) and exhibited context-dependent encoding of the sounds across behavioral contexts (*black bar*). Gray dots represent the percentage of neurons for each session. This figure was compiled using MATLAB 2019, MathWorks software and labeled using Adobe Illustrator CC 2019.

Our ensemble analysis involved using the SSIMS framework to examine the intrinsic similarity of spike patterns associated with the different types of non-instructional auditory stimuli (Passive Listening paradigm) and, second, if MFG and PCG ensemble responses differed for the same auditory stimuli depending on whether the sounds were immediately relevant for action planning, i.e., context-dependent encoding. We used the SSIMS analysis framework to generate relational representations of the PCG (Fig. 6A) and MFG ensembles responses (Fig. 6B) to the non-instructional auditory stimuli from the Passive Listening paradigm and the instructional auditory cues from the Multi-modal tasks (1st cue period from AV and VA trials only). For comparison, baseline ensemble responses from the same set of trials were also included in the representations (Fig. 6A,B, cyan points).

Analysis of the distribution of distances between trials in the SSIMS representations (see Methods for details) revealed that although the PCG ensemble’s response to non-instructional auditory stimuli was not significantly different from baseline (KW test,* p* < 0.01), the MFG ensemble’s responses to the non-instructional auditory stimuli were significantly different from baseline (Fig. 6C,D, white boxplots). Interestingly, despite our findings that both PCG and MFG ensembles exhibited selective responses to the different auditory cues during the Multi-modal task (Fig. 6C,D, grey boxes labelled “Task Cues”; and Fig. 3), neither the PCG nor MFG ensemble’s responses displayed variations in firing patterns for the different non-instructional auditory stimuli (Fig. 6C,D, grey boxplots labeled “Passive stimuli”). Finally, when we examined the effect of task context on the MFG ensemble’s responses to the non-instructional sounds (see Materials for analysis details), we observed that the MFG ensembles exhibited significantly different spiking patterns depending on whether the sounds were immediately relevant for action planning (Fig. 6D, black boxplots).

A single neuron analysis (Fig. 6G,H) revealed that none of the PCG neurons and 24% of the MFG neurons (Fig. 6G,H) responded to the auditory stimuli in the passive listening condition. In line with results from our ensemble analysis, neither the PCG nor MFG neurons exhibited any selectivity for the auditory stimuli during the Passive Listening paradigm (< 1% of neurons across three sessions; *p* < 0.01, KW test). In other words, almost a quarter of MFG neurons exhibited an innate responsiveness to sounds, however, the neurons’ responses did not discriminate between different sounds.

We also examined the effect of task context on the firing rates of individual PCG and MFG neurons by directly comparing responses to sounds in the Multi-modal task (i.e., “red” and “blue” instructional auditory cues) to those observed in the Passive Listening paradigm (i.e., the six different non-instructional auditory stimuli) (see Materials for analysis details). We observed that 45% of the PCG neurons and 51% of MFG neurons exhibited a response modulation to the same sounds depending on the behavioral context (i.e., immediately relevant for action planning versus behaviorally irrelevant). In summary, PCG and MFG neurons only exhibited aural selectivity if a behavioral response was required and only MFG neurons exhibited inherent aural responsiveness to sound in the absence of a behavioral response requirement.

## Discussion

Realizing the full potential of iBCI technology critically depends on continued progress in understanding the neural processes underpinning volitional behaviors, including how the human brain encodes the intent to perform specific actions. The current work investigates the cortical neurophysiology of action planning and volitional intent in two anatomically and functionally distinct motor cortical regions of a person with chronic tetraplegia performing a 2D iBCI task. This study presents the first direct comparison of neural ensemble activity simultaneously recorded in human MFG and PCG.

### MFG: linking sounds to motor plans

Our specific goal was to determine if neurons in MFG contain movement-related signals that are distinct or complementary to those carried in PCG—a region our group and others have explored previously^4,10,13,22,44^. Our results demonstrate that both MFG and PCG neurons are engaged by movement planning and volitional intent; however, differences in the information content and timing of movement-related signals suggests that the two areas perform different types of computations. When robustly engaged, MFG neurons responded to and encoded action-related information before PCG neurons. MFG only displayed action-related information during earlier stages of movement planning, while PCG neurons tended to display action-related information after all necessary cues were provided and a concrete movement plan could be formulated. This suggests that MFG is engaged in an earlier sensorimotor processing stage preceding the appearance of a movement plan in PCG. This conclusion is also supported by the observation that MFG encodes action-related information equally well during the preparation of eye or hand movements, while PCG is more strongly engaged during intended-hand movements than eye movements. Unlike PCG, it appears that MFG may be engaged in movement planning before an effector is determined.

In addition to the timing of activity, the most striking difference between MFG and PCG was revealed when we examined the effect of instructional cue modality: action-related signals were only found in MFG when target information was provided using auditory cues (as opposed to visual cues), while PCG did not exhibit a strong bias towards either cue modality. Our results from the Passive Listening task revealed that, unlike PCG, MFG neurons were inherently sensitive to auditory stimuli; however, selectivity only emerged in MFG when an auditory stimulus was immediately relevant for action planning. Taken together, our results suggest that MFG is engaged in processing auditory information relevant to movement planning that precedes the appearance of a concrete action plan in PCG.

### Comparison with previous work

To guide cortical implantation sites for the two microelectrode arrays used in this study, fMRI was used to locate regions of the cortical surface related to movement planning (MFG site) and execution (PCG site) while excluding areas maximally activated by eye movements. Histologically, human primary motor cortex (Brodmann area 4) is mainly located in the anterior bank of the PCG, gradually transitioning to premotor cortex (PMC, Brodmann area 6) over the crown of the PCG^45–48^. Given the final placement of the microelectrode arrays used for this study, it is likely that our PCG recording site corresponds more closely to area 6. This particular part of area 6 that abuts area 4 has been proposed as a homologue of dorsal premotor cortex (area F2) in the macaque monkey^49^. In support of this conclusion, intended-action signals were observed in PCG before movement onset once an action plan was unambiguously specified, matching results previously reported in monkeys^50^. It is more difficult to draw a direct comparison between our findings in MFG and previous work in primates. However, given the extensive connections between motor and auditory areas, finding aurally responsive neurons in motor regions is not unexpected. Tracing studies in non-human primates^51^ and structural studies in humans^52^ have shown the existence of direct and indirect connections between the posterior regions of the auditory cortex and PMC, frontal eye fields (FEF, area 8), and dorsal prefrontal cortex (PFC, areas 8, 46, and 9). Primate motor electrophysiology studies reported that neurons in the rostral part of the dorsal PMC (area F7)^53^ and ventral PMC neurons respond during sound localization tasks^54^. PMC has also been shown to respond selectively to sounds tied to actions^55,56^. This auditory-motor interaction is apparent in imaging studies of musicians: passively listening to music can activate areas of PMC also activated by playing music^57,58^. Musical training in non-musicians can modulate neural activity in the motor network in response to sound, and PMC is necessary for performing appropriate motor responses to auditory cues^59^.

Our results build upon previous findings by providing the first estimates of the informational content of individual neurons in human MFG during auditory, visual, and mixed sensorimotor transformations, highlighting the specific sensitivity of neurons in this area to auditory stimuli. These findings provide an interesting contrast to similar work examining the informational content of neurons in human anterior intraparietal (AIP) area, which appear to be exclusively selective to visual cues during instructed delay periods, only reflecting task-relevant auditory information after movement initiation^25^. In agreement with previous results obtained from primate M1/PMd single neuron recordings^30^ and human electrocorticography studies^60,61^, our results show that MFG neurons tend to have shorter response latencies than PCG neurons, as is characteristic of a more rostral motor area upstream of PCG. However, the lack of action selective responses in MFG following visual cues, as well as during active iBCI control, differs from previous findings in non-human primate premotor cortex^28,31^.

The presence of chronic cervical spinal cord injury in this participant is a potential confounding factor. Despite mounting evidence that upper motor neuron axonal injury does not preclude voluntary, goal-directed modulation of neurons in the arm-hand area of the PCG^4,10,11,17,22^ it is possible that the activity patterns of non-primary motor areas in the frontal lobe are altered when they are effectively, either directly or indirectly, disconnected from motor outputs. This could also explain the difference between our findings and a previous report from our research team examining single neuron activity in human premotor cortex^39^. Single neuron recordings performed in patients undergoing elective deep brain stimulator (DBS) electrode implantation surgery revealed target-selective PMC activity following visual cues in a task similar to the one employed in the current study (although using only visual cues). Although the participants were undergoing treatment for movement disorders symptoms that were insufficiently responsive to medication (two had Parkinson’s disease and one had essential tremor), they still retained sufficient motor function to perform the task using a digitizing pen and sensor tablet. Another important distinction between the two studies is that the recording sites in the DBS report were considerably medial to the recording site reported here.

### Limitations and future directions

Since the current study was limited to a single participant, we cannot assess inter-subject differences as a potential factor influencing our results. Further opportunities to record from human agranular frontal cortex will be required to understand gradations of functional parcellation of these areas, the variability of functional maps across different people, and potential homology of human MFG to structures in NHPs.

Our results show a clear increase in responsive and selective neural activity in MFG following auditory cues used to formulate movement plans. However, the use of spoken words as instruction cues in the present study does not allow us to rule out the possibility that the observed response selectivity is specific to language processing^49,62^. If this is the case, it is possible that the effects described here could be specific to humans compared to other primates. Recent work suggests that cortical areas traditionally associated with upper limb motion may play a role in speech generation^62^. Further work will be needed to determine if understanding speech also selectively engages MFG and PCG.

It is expected that by recording from multiple functional areas related to motor control, intracortical brain-computer interfaces could be designed to take advantage of a wider variety of information related to intended movement, whether for control of a computer cursor, tablet computer, robotic arm or for the reanimation of a paralyzed limb. The automated feature selection underlying the closed-loop iBCI calibration and decoding approach used here resulted in neural decoders that relied on electrodes which were predominantly from the PCG array. This reflects the lack of signals in MFG related to intended movement direction during volitional control. Future decoding innovations will likely incorporate information about upcoming planned movements, particularly in harnessing data from cortical regions that become less informative during the intended movement. Our findings highlight the importance of considering multiple sensory modalities when evaluating cortical areas for BCI applications and suggest that decoding strategies that differentially engage visual and auditory stimuli could potentially broaden the usability and effectiveness of assistive BCI systems.

## Materials and methods

### Permissions

Permissions for this study were granted by the US Food and Drug Administration (Investigational Device Exemption) and the Institutional Review Boards of Massachusetts General Hospital, the Providence Veterans Affairs Medical Center, and Brown University. All experimental procedures and methods were conducted in accordance with the policies and guidelines associated with these approvals. This study includes one human participant, T10, who gave informed consent and was enrolled in a pilot clinical trial of the BrainGate2 Neural Interface System. The BrainGate2 clinical trial (Identifier: NCT00912041, 03/06/2009) was registered at ClinicalTrials.gov on June 3rd, 2009. CAUTION: Investigational device. Limited by Federal law to investigational use.

### Participant

Participant T10 is a right-handed man, 35 years old at the time of this study, with tetraplegia due to a cervical C4 AIS-A spinal cord injury that occurred nine years before enrollment in the BrainGate2 clinical trial. The participant had two surgically-placed, 96-channel microelectrode arrays with 1.5 mm electrodes (Blackrock Microsystems, Salt Lake City, UT), one in the "hand knob" area of the left precentral gyrus (PCG)^38^, and the other in the caudal region of the left middle frontal gyrus (MFG).

### Structural and functional MRI Protocol: selection of array locations

Structural and functional MRI mapping protocols were performed two months prior to array placement to identify functionally relevant cortical regions. A hybrid block and event-related paradigm consisting of an “Object reaching and grasping” task with an instructed delay period was used to identify cortical regions engaged during upper-limb movement planning and attempted movement execution (Supplementary Fig. S4). A separate “Eye movement” task with an instructed delay period was used to identify cortical areas engaged during the planning and execution of eye movements in the absence of upper-limb movements (See Supplementary Materials and Fig. S4 for more details on fMRI protocol and the selection of the final array location descriptions).

During the surgical procedure, the final location of the array targeting the upper-limb movement execution was selected to be as close as possible to the corresponding peak activation site while ensuring that it was placed in a mechanically stable position near the crown of the gyrus, devoid of cortical vessels (Fig. 1 and Figs. S6, S7). The final location of the array targeting upper-limb movement preparation was selected to be as close as possible to the corresponding peak activation site, given the same surgical constraints and away from the FEF region (Supplementary Figs. S6, S7).

### Electrophysiological signal acquisition and pre-processing

The signals from each array were analog filtered between 0.3–7500 Hz then sampled at 30 kHz by two Neuroport Neural Signal Processors (Blackrock Microsystems, Salt Lake City, UT). Custom code on a real-time computer running Simulink Real-Time Operating System (MathWorks, Natick, MA) was used to pre-process the signals, starting with down-sampling from 30 to 15 kHz. Refer to Supplementary Materials for details on channel selection, feature extraction, and decoder calibration. Differences in spike waveform shape and amplitude were used to identify single unit activity using custom-made software^63^, and then manually inspected for consistency (OfflineSorter, Plexon Inc., Dallas, TX). All analyses of array data were performed on single unit spike rates. For simplicity we referred to well isolated single units as ‘neurons’ throughout the paper. Single units and trial numbers for the iBCI tasks are summarized in Table 2.

**Table 2:** Summary data for single unit and trial numbers during iBCI tasks.

	Eye-hand iBCI task			Multi-modal cues iBCI task		
	MFG units	PCG units	Number of trials	MFG units	PCG units	Number of trials
Session 1	52	64	153	72	56	267
Session 2	62	67	185	73	58	470
Session 3	37	63	131	51	61	273

MFG, middle frontal gyrus; PCG, precentral gyrus; iBCI, intracortical Brain Computer Interface.

### iBCI Multi-modal cues task

The participant performed the Multi-modal Cues task (Fig. 2) on post-implant trial days 356, 357, and 362. We used an instructed-delay, center-out task paradigm with Multi-modal target cues to determine if MFG is biased towards processing multi-modal or unimodal movement-related cue information. The task required the participant to direct a neurally controlled cursor from the center of the screen to the correct of four targets located radially on the screen. Target selection required the participant to integrate an auditory cue (“red” or “blue” lexical audio cue) and a visual cue (circle or square shape) in order to determine the identity of the correct target. Each target identity was defined by unique combinations of a color (red or blue) and a shape (circle or square), such that the different audio-visual cue combinations described the identity of the correct target (e.g., “red” audio cue + circle visual cue indicated that a red circle was the correct target). The targets had a cardinal alignment and were placed in a clockwise manner as a red circle, red square, blue square, blue circle for all trials in sessions 1 and 3, and blue square, red square, blue circle and red circle in all trials for session 2.

The auditory and visual cues were presented during the instructed delay period in either a sequential manner (AV, audio cue first trials; VA, visual cue first trials) or a simultaneous manner (A + V, simultaneous audio and visual trials). The AV trials began with the presentation of the neurally-controlled cursor at the center of the screen and the four targets radially located around the cursor (Fig. 1A). After a variable delay of 1–2 s (baseline phase), an auditory cue was presented for a delay-period of 1–2 s (partial-information delay phase) before the visual cue was flashed at the center of the screen. After a further delay of 1–2 s (full-information delay phase), a dimming of the central target, an auditory ‘beep’ (1047 Hz), and a brightening of the centrally-located cursor indicated the participant to use movement-imagery to direct the cursor to the cued target within 5 s and to maintain it there for a dwell duration of 0.3 s. The cursor position was fixed at the center of the screen up until the go signal to minimize any neural responses that might result from efforts in stabilizing the cursor throughout the delay period. In the VA trials, the presentation order of the auditory and visual cues was reversed (Fig. 1B). In A + V trials, both cues were presented simultaneously (i.e., no partial-information delay phase) at a variable delay of 1–2 s before the go signals (described above) (Fig. 1C). In all trial types, auditory cues lasted on average 320 ms, and visual cues were presented for 330 ms. Audio cues originated from two speakers located on the cart to which the task screen was anchored. *Unsuccessful trials* and trials with *false starts* were excluded from data analysis (See Supplementary Materials for details on Trial Rejection).

### Passive listening task

The participant started and ended the research session with the Passive Listening task on post-implant trial days 357 and 362. The task displayed a fixation cross at the center of the screen. Each trial lasted 1–2 s. During this time, the participant was presented with the auditory stimuli; no behavioral response was required. The auditory stimuli were a mix of colors (e.g., the word “red”), cardinal directions, or pure tones (i.e., non-lexical) and were presented pseudorandomly. The ‘color’ based auditory cues were made up of auditory stimuli that were either previously relevant during the Multi-modal Cues task (e.g., “red” or “blue”) or were irrelevant under any task context (e.g., “pink”). Similarly, the 1047 Hz pure tone stimulus was previously relevant during the Multi-modal Cues task as a go signal, while the 523 Hz tone was irrelevant under any context. A different set of auditory stimuli were used between sessions 1 and 2 (red, pink, north, south, 523 Hz tone, 1047 Hz tone) and session 3 (red, blue, pink, north, square, circle). The “red” auditory stimulus appeared in all sessions for the Passive Listening task and the Multi-modal task.

### “Responsive” versus “selective” neurons

A neuron was tested for responsiveness to cues by comparing activity within the baseline phase and the activity in a sliding window (shifted in 20 ms steps). The baseline distribution consisted of the time-averaged spike rates within the 300 ms window preceding cue onset for each trial. Likewise, the neurons’ spike rates were time-averaged across a 300 ms sliding window for each trial and grouped by the cued target. The baseline and grouped distributions were tested for significance using the KW test (p < 0.01). A neuron was tested for selectivity to the different actions by grouping trials according to the cued target and comparing their activity within a common window (KW p < 0.01). Specifically, a 300 ms window was shifted in 20 ms steps across a transition of interest (e.g. delay phase to go phase).

### Ensemble spike-train similarity space analysis (SSIMS)

SSIMS is an unsupervised dimensionality reduction algorithm designed to capture the intrinsic relationships between spiking responses on a trial-by-trial basis^42^. The algorithm begins by calculating pairwise distances between spiking patterns using the metrics developed by Victor & Purpura^42^. This approach is based on the concept of ‘edit distances’ where one spike train is transformed into another using a series of operations: adding a spike, deleting a spike, or moving a spike in time. The first two operations are assigned a ‘cost’ equal to 1, while moving a spike in time is assigned a cost equal to qΔt, where Δt represents the duration of the time shift and q is a parameter that determines the temporal precision of the metric. For the results presented here, q was set so that shifting a spike by more than 100 ms was equivalent to deleting the spike and then inserting another spike. The second part of the SSIMS algorithm uses t-SNE^64^ to reduce the dimensionality of the concatenated pairwise distance matrices for each neuron in the ensemble, resulting in a map that preserves local neighborhood structure. Each point on the SSIMS map corresponds to a trial, and the distance between the trials indicates the degree of similarity between the corresponding spiking patterns. SSIMS was applied to spike train data from all MFG or PCG neurons occurring in a 500 ms window, beginning 200 ms after cue presentation for MFG and 400 ms for PCG.

A Euclidean distance analysis was used to quantify the degree of clustering between different categories of trials in each of the SSIMS maps: the two audio cues, two visual cues, or the four audiovisual cue combination indicating each of the targets. A single box plot (Figs. 3 and 6) shows the distance distribution for pairs of trials spanning different trial categories, i.e., the “Between-category” distances. The “Between-category” distribution was normalized against the median distances among all pairs of trials belonging to a common category, i.e., “Within-category” distribution. A “Between category” data-point was equal to 1 when it had the same separation as the “Within-category” median and a value greater than 1 when the separation between trials from different categories was larger than the separation between trials from a common category.

### Ensemble prediction using an SVM

Neural ensemble information was assessed with multiple methods, including Naive Bayes and Support Vector Machines (SVMs). Although our findings were similar across the different classifiers, previous studies have reported that SVM classifiers tend to outperform other classifiers when decoding a given feature from an ensemble containing both feature-selective and non-selective neurons^65^, therefore, results were reported for the SVM method, only. The linear SVM (MathWorks Statistics and Machine Learning Toolbox) used five-fold cross-validation and the error-correcting output codes (ECOCs) multiclass framework. Classification was performed on a 300 ms window shifted in 20 ms steps across the transition of interest (e.g., delay phase to go phase). For each window, the mean rates (features) and associated targets (labels) were used to compute classification accuracy. Confidence intervals were determined by randomly permuting the labels 10,000 times.

### Single neuron response onset latency

The response onset latencies of MFG and PCG neurons were estimated by computing the distribution of spike-counts in the baseline phase, then finding the earliest time at which the post-cue distribution of spike-counts deviated significantly from baseline.

For a given neuron, a baseline distribution was generated by counting the number of spikes in each consecutive and non-overlapping 100 ms bin within a -800 ms to 0 ms pre-cue window. Similarly, spike-count distributions were generated for each 100 ms bin in the 0 ms to 800 ms post-cue window, using 20 ms time-steps. We did not analyze beyond 800 ms post-cue in order to avoid any activity that may be anticipatory of upcoming cues. A KW (*p* < 0.01) was carried out between the baseline distribution and each post-cue distribution for a given neuron. Response onset was defined by observing three consecutive bins with significant *p*-values^66^. The mid-point of the first bin was selected as the response onset latency for that neuron. Estimated response onset latency values of less than 40 ms were excluded from the analysis^67^.

This procedure was carried out separately for each cue type in the Multi-modal task trials, i.e., the two auditory cues in the AV trials, the two visual cues in the VA trials, and the four audio-visual cues in the A + V trials, and the four auditory cues in the Eye-Hand task. This avoided the possibility of averaging-out a given neuron’s evoked response if the neuron was only selectively active for a subset of cues. The shortest latency across each of the cue types was recorded as the neuron’s response onset latency. In this fashion, a single latency was reported for each responsive neuron in each condition of the three trial types in the Multi-modal task (AV, VA, and A + V). Latency data points were pooled across each of the three sessions for a given trial type, which included response onset latency values from an average of n = 20 (AV), 13.6 (VA), and 32.3 (A + V) PCG neurons per session and n = 41.6 (AV), 20.6 (VA), 34.3 (A + V) MFG neurons per session. A KW test was used to assess if there was a significant difference between the MFG and PCG latency distributions during a given task and if there was a significant difference between the latency distributions of a given neuronal ensemble (i.e., MFG or PCG) across tasks. The median across-task MFG and PCG response onset latencies were estimated by pooling data across all sessions and all tasks.

### Passive listening versus Multi-modal task data analysis: contextual encoding of auditory stimuli

We compared the proportion of MFG and PCG neurons that were responsive/selective to auditory stimuli during the presentation of the behaviorally relevant auditory stimuli (Multi-modal task) or behaviorally irrelevant auditory stimuli (Passive Listening task).

A neuron’s aural responsiveness was assessed by comparing the baseline and stimulus-evoked firing rates for each of the presented auditory stimuli/cues. For a given stimulus, separate baseline and stimulus-evoked distributions were generated by counting the number of spikes in a 500 ms window before and after cue onset for all trials. The post-cue window was 200 – 700 ms and 400 – 900 ms after cue onset for MFG and PCG neurons, respectively. The difference in the MFG and PCG windows ensured that the window was centered around the peaks of MFG and PCG activity (for more on response onset latencies, see Results). A neuron was considered aurally responsive if, for any of the auditory stimuli in each task, a KW test (*p* < 0*.*01) revealed a statistically significant difference in activity between the baseline and post-cue phases. A Bonferroni correction was made to the significance threshold to compensate for multiple comparisons. Each neuron’s aural selectivity was assessed using stimulus-evoked response distributions similar to that described in the aural responsiveness test (i.e., calculating spike counts in 500 ms window during the response period). A neuron was considered aurally selective if a KW test (*p* < 0.01) revealed a statistically significant difference in activity between responses to the different auditory stimuli within a given task (n = 2, Multi-modal groups; n = 6, Passive Listening groups).

To assess the effect of task context on each neuron’s responses to the auditory information (i.e., context-dependent response modulation), we compared the Multi-modal stimulus-evoked responses for a single cue (e.g., "red" or "blue" auditory cue) to the Passive Listening stimulus-evoked responses to all auditory stimuli (i.e., all six trial types in the passive listening task were grouped together for each neuron). We elected to group the different stimulus trial types in the passive listening task into a single group for this analysis as our previous examination revealed that the neurons did not exhibit any significant response differences for the various auditory stimuli in this task (Fig. 6 C and D; gray boxplot). Stimulus-evoked spike-count distributions were compared in a similar manner to that described in aural responsiveness test, (i.e., by calculating spike counts in 500 ms window during response period for each trial). A neuron was considered differentially modulated between the two tasks (i.e., task-dependent modulation) if a KW test (*p* < 0*.*01) showed a statistically significant difference between the two distributions of spike counts. A Bonferroni correction was made to the significance threshold to compensate for multiple comparisons. Example PCG and MFG single neuron responses during the Passive Listening and Multi-modal cues are from trial day 356, with PCG neurons from (Fig 6E, L-to-R) channel 83 unit 1, channel 66 unit 2, channel 55 unit 1, and channel 2 unit 1. Example MFG neurons (Fig 6F, L-to-R) are from channel 67 unit 1, channel 17 unit 1, channel 68 unit 1, channel 33 unit 1.

## Supplementary information

Supplementary Information 1.

## Data Availability

All reasonable requests for collaboration involving materials used in the research will be fulfilled provided that a written agreement is executed in advance between MGH and the requester (and his or her affiliated institution). Such inquiries can be directed to L.R.H.
