# Supplementary material for: Auditory cues reveal intended movement information in middle frontal gyrus neuronal ensemble activity of a person with tetraplegia
Source: Sci Rep. 2021 Jan 11;11:98. doi: 10.1038/s41598-020-77616-8 (PMC7801741; doi:10.1038/s41598-020-77616-8)
Supplement: Supplementary file 1 — Supplementary Information 1. [file 41598_2020_77616_MOESM1_ESM.pdf]

SCIENTIFIC REPORTS
natureresearch

# Supplementary Materials

## Auditory cues reveal intended movement information in middle frontal gyrus neuronal ensemble activity of a person with tetraplegia

Tommy Hosman*, Jacqueline B. Hynes*, Jad Saab*, Kaitlin G. Wilcoxen, Bradley R. Buchbinder, Nicholas Schmansky, Sydney S. Cash, Emad N. Eskandar, John D. Simeral, Brian Franco, Jessica Kelemen, Carlos E. Vargas-Irwin♦ and Leigh R. Hochberg♦.

* Co-first authors, contributed equally.

♦ Corresponding authors, contributed equally: Carlos_Vargas_Irwin@brown.edu and leigh_hochberg@brown.edu

**This document includes:**

Supplementary Analysis
Fig. S1. In contrast to PCG, MFG lacked consistent action selective activity patterns in a visually guided 2D center-out target acquisition task.
Fig. S2. Example MFG single neuron activity during the Eye-Hand center-out iBCI task sequence.
Fig. S3. MFG neurons are responsive and selective before PCG neurons in an instructed delay task.

Materials and Methods
Fig. S4. fMRI task structure for Object Reach and Eye Movement paradigms
Fig. S5. Representative statistical maps of fMRI task activation from the voxel-based analysis.
Fig. S6. Statistical maps of fMRI task activation in the left cerebral hemisphere from the surface-based analysis.
Fig. S7. Surface mapping of upper-limb movement execution, movement preparation, and FEF activation foci.
Table S1. Number of neural features from each array used to control iBCI cursor across sessions.

# Supplementary Analysis

**Preliminary analysis of MFG and PCG during a visually guided iBCI center-out task** We used a 2D center-out task with 8 uniformly spaced targets (Supplementary Fig. S1A, B) in our preliminary examination of neural activity in MFG and PCG related to intended movements. At the beginning of each trial, the cursor was reset to a central target. A change of color in one of the peripheral targets was used as an instruction cue. After a variable delay a white circle appeared around the center target, serving as a “go” cue that indicated to the participant to move the cursor towards the target using neural control. There were two types of trials: for instructed delay (ID) trials (Supplementary Fig. S1A), the time between the instruction cue and the go cue was uniformly distributed between 1.5 and 2.5s. For trials without instructed delay, the instruction and go cue were provided simultaneously (Supplementary Fig. S1B). Note that in this task, all cues provided to the participant were visual (in contrast to other tasks described in this work, where both visual and auditory were used). We characterized the activity of single neurons as being “responsive” or “selective”. Neurons that displayed significantly different firing rates during either the Delay or Go epochs compared to baseline were labeled as “responsive” (KW, $p<0.01$). Neurons that also had distinct changes in firing rate for at least one of the possible targets (KW, $p<0.01$) were considered “selective”. Firing rates were binned using a sliding 300 ms time window, shifted in 20 ms increments.

For the visually guided iBCI center-out task, PCG neurons displayed robust responsive and action selective activity patterns, starting during the instructed delay and extending into the active target acquisition phase (Supplementary Fig. S1C, D; teal traces). MFG neurons displayed markedly different properties (Supplementary Fig. S1C, D; yellow traces). While neurons in this area also responded during the task – in that they displayed firing rates above baseline – there was a marked lack of action selective information compared to PCG. Note that due to the lack of information related to intended movement detected in MFG during this task, neural control of the cursor relied

on PCG signals (see Supplementary Materials and Methods for details on decoder calibration).

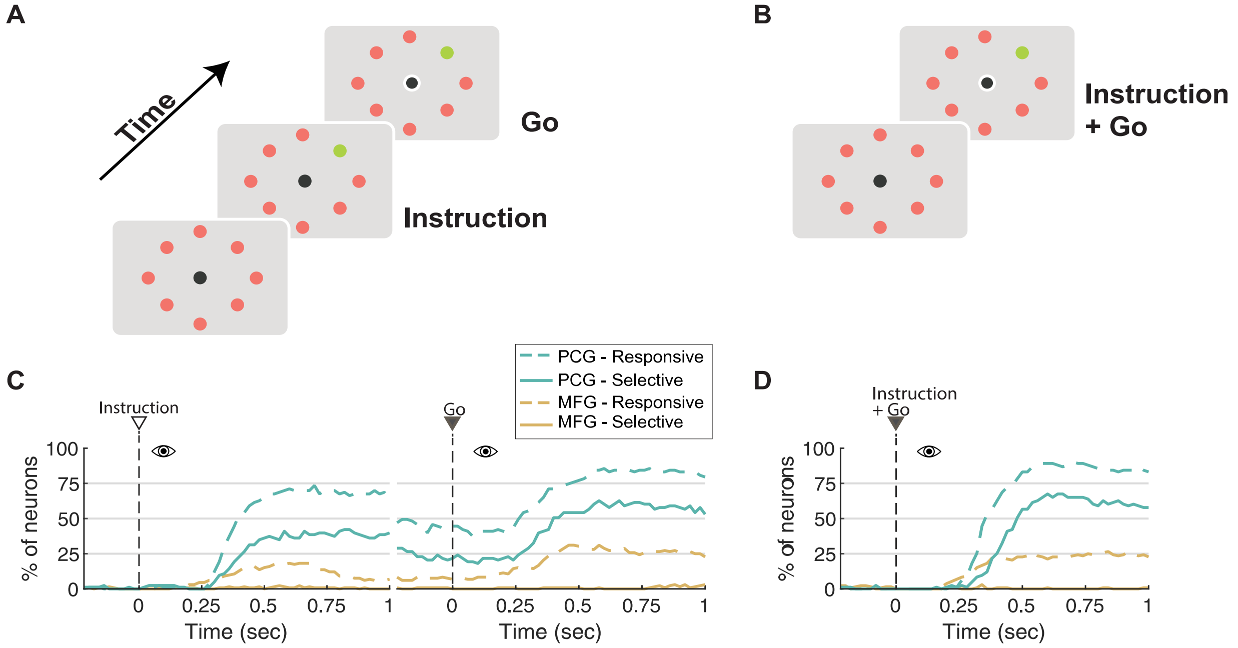


**Fig. S1. In contrast to PCG, MFG lacked consistent action selective activity patterns in a visually guided 2D center-out target acquisition task.**

**(A)** Visually guided center out task sequence with instructed delay (ID trials). A white circle appeared around the center target as the "go" cue. (**B**) Task sequence for trials without an instructed delay (no instructed delay, NID). (**C**) Proportion of responsive (broken lines) and selective neurons (solid lines) for ID trials. Eye icon indicates presentation of visual cue. (**D**) Proportion of responsive and selective neurons for NID trials. This figure was compiled using MATLAB 2019, MathWorks software, and Adobe Illustrator CC 2019.

**iBCI Eye-Hand task description** The Eye-Hand iBCI center-out task was designed to investigate the extent to which neurons in the implanted middle frontal gyrus (MFG) and precentral gyrus (PCG) were engaged during the planning and execution of eye movements versus the planning and (attempted) execution of hand movements.

The participant performed the Eye-Hand task on post-implant days 328, 337, and 342. The 2D target selection task required the participant to acquire one target (highlighted in red) of four radially arranged targets surrounding a circle at the center of the screen (Supplementary Fig. S2A, B). An alternating block-design task was used to separately examine neural activity related to intended hand-movement in the absence of eye movements ("hand-control" trial blocks) and eye movement related neural activity in the absence of intended hand-movements ("eye-control" trial

blocks). We note that the task nomenclature, i.e., "hand-control" and "eye-control", refers to the nature of the underlying method used to control the target acquisition system, in that the targets were acquired using either directed eye movements (eye-control) via an infrared eye tracker, or a neurally-controlled cursor via the iBCI, which was driven by attempted hand movements (hand-control).

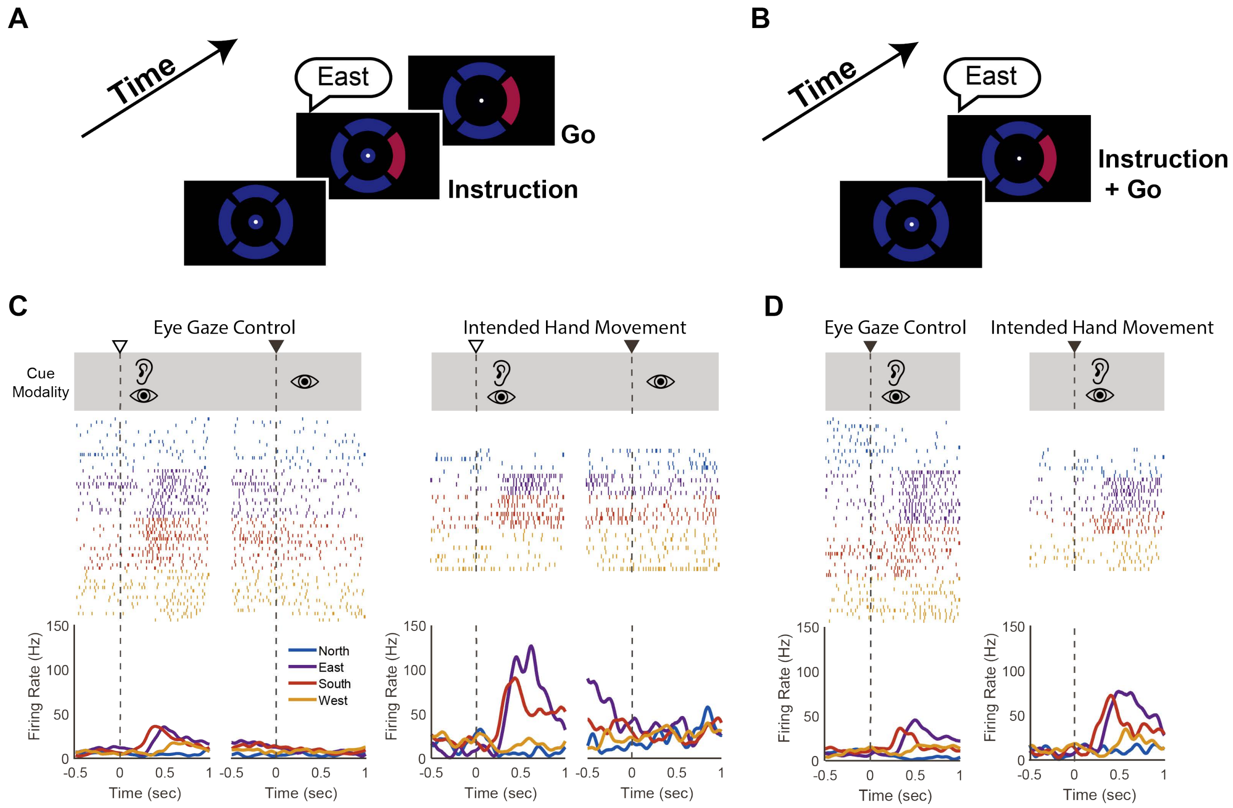


**Fig. S2. Example MFG single neuron activity during the Eye-Hand center-out iBCI task sequence.**

**(A)** Eye-Hand task sequence with instructed delay (ID trials). The center blue circle dimmed as the "go" cue. (**B**) Eye-Hand task sequence without an instructed delay (no instructed delay, NID). (**C**) Example raster-histograms for a single MFG neuron during ID trials in the eye-control condition (left) and hand-control condition (right). This neuron exhibited a larger response before intended hand movements and was selective for particular directions (south and east). Cue presentations (Instruction or Go) are indicated by vertical dashed lines; white arrowhead represents the instruction cue, with eye and ear icons indicating the presentation of a visual cue and auditory cue, respectively; black arrowhead represents the go cue (dimmed blue central circle). Example neuron from trial day 337, MFG array channel 29, unit 1. **(D)** Example raster-histograms for NID trials for the same MFG neuron as in (**C**) showing similar modulation to eye and intended hand movements. Histogram traces show the mean firing rates of the given neuron for each target (200ms Gaussian smoothing window). This figure was compiled using MATLAB 2019, MathWorks software, and Adobe Illustrator CC 2019.

During the hand-control blocks, the participant was required to maintain fixation within 4.3 cm of a centrally located circle (i.e., fixation window) while directing a neurally-controlled cursor that was driven by intended hand movements (hand-cursor) from the central circle to the correct of four radially-spaced arc targets (Supplementary Fig. S2A). During the eye-control blocks, the participant was required to constrain the hand-cursor within 3 cm (~ 3 visual degrees) of the central circle while directing eye-gaze from the central circle to the correct of four radially-spaced arc targets. Eye position was tracked during both task types using an EyeTribe eye-tracker and calibrated using Tobii calibration software. The 4.3 cm fixation threshold used in the hand-control blocks was chosen to accommodate a baseline jitter in the participant's disconjugate gaze (see Data Analysis - Trial Rejection for more details on cursor/eye movement thresholds). The participant was verbally reminded of the upcoming block type at the end of each block of trials.

Both the eye-control and hand-control blocks had trials with and without an instructed delay. An instructed-delay trial began with the presentation of a blue circle and the hand-cursor at the center of the screen, along with four blue radially-spaced arc targets (Supplementary Fig. S2A). After a variable wait period of 2-3 seconds (baseline phase), one of four targets was cued through both a change of color from blue to red and an audio announcement of its cardinal direction (i.e., "north", "south", "east", or "west"). Simultaneous auditory and visual instructions were used. After a variable delay of 1-2 seconds ("delay phase"), the central circle dimmed, cueing the participant to direct either the hand-cursor, when in a hand-control block, or eye position, when in an eye-control block, to the cued target (go phase). In the eye-control blocks, the target was considered acquired if eye position reached the target in under 5 seconds and was maintained within 2.2 cm of the cued target for a dwell duration of 0.3 seconds. In the hand-control blocks, the target was considered acquired if the hand-cursor reached the cued target in under 5 seconds and was maintained within the target for at least 0.3 seconds. The movement of the hand-cursor during eye-control was attenuated by 60% to reduce task difficulty. For trials without an instructed delay, both target cues and the go cue were presented simultaneously (Supplementary Fig. S2B). Trial types and targets were presented pseudo-randomly, and trial intervals were varied, ensuring an equal distribution of

target locations in each trial type and minimizing the ability to precisely predict the temporal onset of the next cue. Catch trials, where no go cue was provided, were also included to reduce the motivation to predict the timing of an upcoming go cue. Under both block conditions, *unsuccessful trials*, trials with *insufficient fixation*, and trials with *false starts* were excluded from the analysis (See Materials & Methods – Trial Rejection).

Visual feedback was provided to the participant about eye-position whenever his gaze fell within any of the four peripheral targets (target changed to yellow color) to reinforce task success. Visual feedback was also provided to the participant whenever the hand-cursor strayed outside the permitted 3 cm boundary around the central circle during the eye-control blocks (central circle changed to yellow color). The visual feedback about the hand-cursor served to remind the participant to perform the task with his eyes rather than with intended hand movement.

**iBCI Eye-Hand task data analysis: Single neuron and ensemble analysis** We characterized the activity of single neurons in the Eye-Hand task as being "responsive" or "selective" using the same procedure described for the visually guided center-out task. The PCG array consistently displayed a larger fraction of both responsive and selective neurons during hand-control blocks compared to the eye-control blocks (Supplementary Fig. S3A-D; teal solid lines above teal dashed lines). During the instructed delay, the peak number of responsive neurons in PCG was ~7% and ~23% for the eye-control and hand-control blocks, respectively (Supplementary Fig. S3A). The number of selective PCG neurons for this time period was ~3% in eye-control blocks, with a more than four-fold increase in hand-control blocks (~15%) (Supplementary Fig. S3C). A similar pattern was observed during the go phase following the instructed delay: ~19% of neurons were responsive in eye-control blocks and up to ~35% in hand-control blocks, while ~3% of neurons were selective in eye-control blocks and ~17% in hand-control blocks. The go phase of NID trials (with target and go cues presented simultaneously) displayed similar numbers of selective and responsive neurons to those observed during the go phase of instructed delay trials (Supplementary Fig. S3B,D; teal solid lines above teal dashed lines).

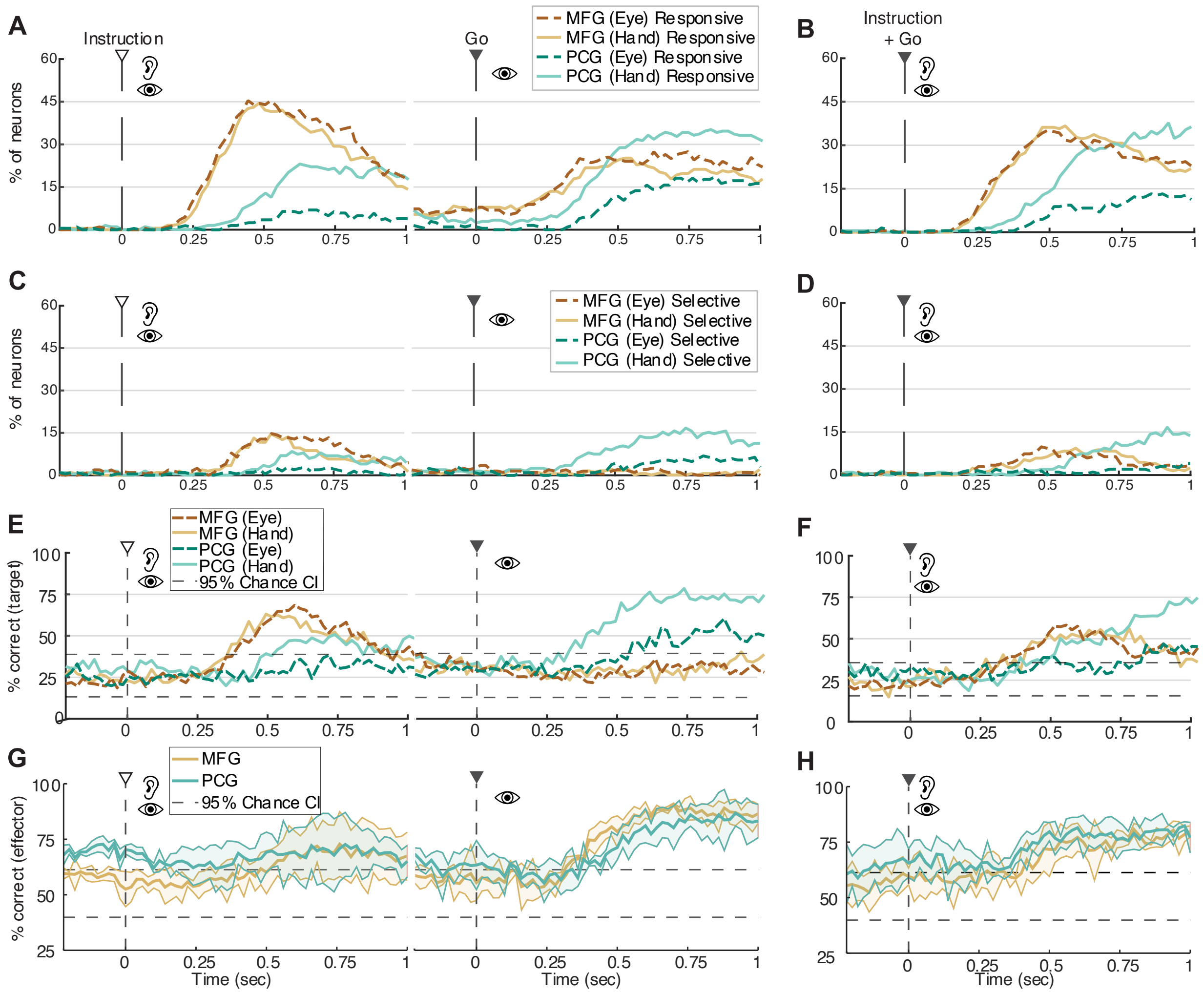


**Fig. S3. MFG neurons are responsive and selective before PCG neurons in an instructed delay task.**

Proportion of responsive (**A**) and selective (**C**) MFG and PCG neurons in the instructed delay (ID) trials of the Eye-Hand task. Proportion of responsive (**B**) and selective (**D**) MFG and PCG neurons in the Eye-Hand trials without an instructed delay (NID). (**E**) Target classification accuracies using MFG and PCG neurons in the ID trials of the Eye-Hand task. (**F**) Target classification accuracies using MFG and PCG neurons in the NID trials of the Eye-Hand task. (**G**) Two-way classification of control effector (eye or hand) using MFG and PCG neurons in the ID trials, and (**H**) the NID trials of the Eye-Hand task. Eye and ear icons indicate presentation of visual cue and auditory cue, respectively. This figure was compiled using MATLAB 2019, MathWorks software and labeled using Adobe Illustrator CC 2019.

We used predictive decoding models to assess the informational content of neuronal ensembles in MFG and PCG. A multiclass support vector machine (SVM) with five-fold cross-validation was used to predict the cued target (i.e., intended action) for each trial based on the activity of all neurons in each area within a sliding 300ms window (Supplementary Fig. S3E, F; See Materials & Methods for classifier details).

Decoding accuracy was consistently higher for hand than eye trials in PCG (Supplementary Fig. S3E, F; teal solid lines above teal dashed lines). During the instructed delay phase, the intended action was decoded with up to 50% accuracy for hand trials (25% chance) and remained within expected chance levels for eye trials (Supplementary Fig. S3E). For both ID and NID go phases (Supplementary Fig. S3E, F), peak decoding accuracy exceeded chance-levels for both hand and eye trials but was consistently higher for hand trials (75%, 78% hand vs 47%, 60% eye). In contrast to PCG, MFG ensemble classification accuracies were nearly identical for both eye and hand-control, consistent with single neuron analysis (Supplementary Fig. S3E, F; solid and dashed brown lines). During the instructed delay, ensemble decoding accuracy peaked around 68% (eye), and 63% (hand). Following go cues, classification trends paralleled those seen in single neuron results: In trials with instructed delay, classification accuracy remained within expected chance levels following the go cue; however, in trials without instructed delay, accuracies increased above chance levels during the go phase, with a peak ~50% (Supplementary Fig. S3F).

Our results suggested that MFG, but not PCG, displayed consistent activity patterns independent of the engaged effector (eye or hand). In order to test this hypothesis more directly, we performed an additional decoding analysis that specifically attempted to classify whether a given 300ms sliding time window belonged to an eye or hand control block based on neural ensemble activity (Fig. 3G, H). Surprisingly, both MFG and PCG yielded classification accuracy above expected chance levels during the delay and go phases (with peaks at 70 and 80%, respectively). This finding suggests that, even though similar proportions of MFG neurons are engaged for eye and hand control blocks, the ensemble firing patterns differ in each case. The availability of this information in MFG in advance of PCG could yield opportunities for explicitly incorporating this information into future real-time neural decoding algorithms.

# Materials and Methods

**Structural and functional MRI Protocol** Structural and functional MRI mapping protocols were performed two months prior to array placement to identify functionally relevant cortical regions. A hybrid block and event-related paradigm consisting of an "Object reaching and grasping" task with an instructed delay period was used to identify cortical regions engaged during upper-limb movement planning and attempted movement execution (Supplementary Fig. S4). A separate "Eye movement" task with an instructed delay period was used to identify cortical areas engaged during the planning and execution of eye movements in the absence of upper-limb movements (Supplementary Fig. S4).

Imaging was performed on a Siemens 3T TIM Trio scanner using a 12-channel Head Matrix coil (Siemens Healthcare, Erlangen, Germany). Command-mode, fixed volume, mechanical ventilation was maintained throughout imaging, matched to the participant's home portable ventilator settings. High resolution sagittal T1-weighted images were acquired before and after intravenous gadolinium contrast administration. Non-contrast-enhanced images were optimal for visualization of the cortical surface. Contrast-enhanced images were used to visualize cortical veins (Fig. 1A). Each acquisition used a 3D-multiecho-MPRAGE sequence (T1-MEMPRAGE) with isotropic resolution 1 $mm^3$, matrix 256 x 256 in-plane x 208 slice partitions, TE 1.64/3.5/5.36/7.22ms, TR 2530ms, TI 1200ms, flip angle 7°, iPAT acceleration factor 2, producing a single composite root-mean-square T1-weighted image volume. High resolution sagittal T2-weighted images were acquired using a 3D-SPACE sequence, with in-plane resolution 0.49 x 0.49 $mm^2$, slice thickness 1 mm, in-plane matrix 256 x 256 with zero-fill interpolation to 512 x 512, 192 slice partitions, TE 402ms, TR 3200ms, flip angle 120°, iPAT acceleration factor 2. High resolution sagittal T2-weighted fluid-attenuated inversion recovery (FLAIR) images were acquired using a 3D-SPACE sequence, with in-plane resolution of 1.0 x 1.0 $mm^2$, slice thickness 1 mm, in-plane matrix 256 x 256, 192 slice partitions, TE 355ms, TR 5000ms, TI 1800ms, flip angle 120°, iPAT acceleration factor 2. BOLD fMRI images were acquired using an echo-planar

gradient-echo T2*-weighted sequence, in-plane resolution 3 x 3 $mm^2$, slice thickness 3 mm, interslice gap 0.75 mm, interleaved, TE 30ms, TR 3000ms, 36 slices, 126 brain volumes per run.

**fMRI instructed delay Object Reach and Eye Movement tasks** A hybrid block and event-related paradigm consisting of an "Object reaching and grasping" task with an instructed delay period was used to identify cortical regions engaged during upper-limb movement planning and attempted movement execution (Supplementary Fig. S4). A separate "Eye movement" task with an instructed delay period was used to identify cortical areas engaged during the planning and execution of eye movements in the absence of upper-limb movements. For brevity, these fMRI task paradigms will be referred to as the "Object Reach" and "Eye Movement" tasks, respectively, noting that they both incorporate an instructed delay.

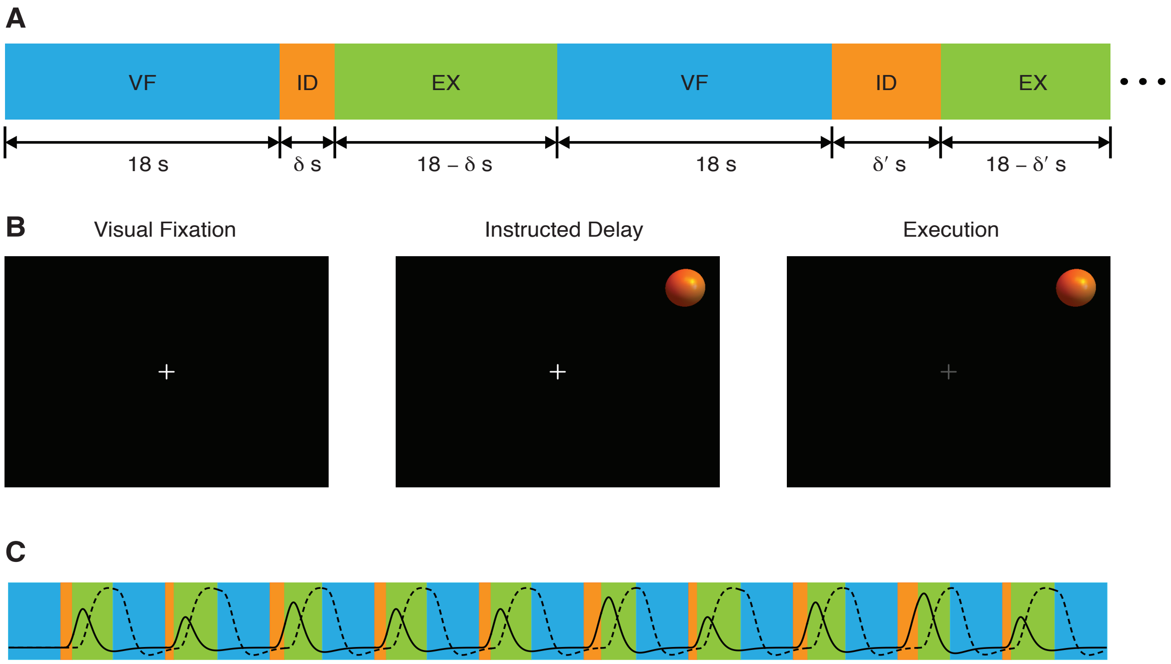


**Fig. S4. fMRI task structure for Object Reach and Eye Movement paradigms.**

**(A)** Hybrid block and event-related paradigm, consisting of three cyclic conditions: visual fixation baseline (VF), instructed delay (ID), and execution (EX). VF block duration was 18 s. ID block duration, $\delta$, was pseudorandomized between 3 s and 7 s. EX block duration was 18 – $\delta$ s, where $\delta$ was the length of the preceding ID block. Therefore, each combined ID-EX duration was 18 s, matching the length of the VF blocks, and each VF-ID-EX cycle was fixed at 36 s. **(B)** Visual stimuli for the VF (left), ID (center), and EX (right) blocks. During the VF blocks, the participant fixated on a central white cursor. At the onset of each ID block, an orange sphere appeared in a pseudorandomized corner of the screen; the participant was instructed to continue fixation on the

white cursor and to wait for an upcoming GO cue. The GO cue, indicating the onset of the EX block, was dimming of the white cursor from the preceding ID block. In the Object Reach task, the participant was instructed to attempt to repetitively reach out for the sphere with his right hand, grasp it, move it to the center cursor, and release it, while continuing to fixate on the dimmed/gray cursor. In the Eye Movement task, he was instructed to repetitively shift his gaze back and forth between the central gray cursor and the sphere as though he was repeatedly dragging the sphere to the center with his eyes. Simultaneous disappearance of the sphere and reversion of the cursor from gray to white indicated onset of the next VF block. **(C)** Paradigm schematic for a full run, consisting of 10.5 VF-ID-EX cycles, 126 samples at 3 s intervals (TR), lasting 6.3 min. Solid and dashed curves represent the relative hemodynamic response models for the ID and EX contrasts, respectively, which were the main regressors in the GLM statistical analysis of the BOLD fMRI signal. Covariance between these two regressors, due to their intrinsic sequential timing, reduces the efficiency with which the responses to the two stimuli are estimated in the GLM. The efficiency is somewhat improved by jittering the durations of the ID and EX blocks. Image (**A**) was created in Adobe Illustrator CC 2019, image (**B**) was created in Mathematica 10.3 for MacOS, and image (**C**) was created in Mathematica 11.1 for Mac OS.

The Object Reach task consisted of three cyclic conditions: visual fixation (VF), instructed delay (ID), and execution (EX) (Supplementary Fig. S4A, B). The VF condition was intended to represent a state of relative rest. The ID condition was intended to represent a state of preparation immediately preceding motor execution, whereas the EX condition was intended to represent a state of attempted motor execution. The conditions cycled sequentially through VF (18 s duration), ID (3 - 7s duration), and EX blocks (11 - 15 s duration). The onset of the VF block was marked by the appearance of a centrally located white cursor, which was then followed by the additional appearance of an orange sphere in one of the corners of the field of view during an ID block.

The participant was instructed to maintain fixation on the central white cursor throughout the VF and ID blocks until the dimming of the central cursor from white to gray during the EX block. The dimming acted as a "go" signal, indicating to the participant that they should begin repetitively attempting to reach out for the ball with their right hand, grasp it, move it to the center cursor, and release it. The participant was instructed to perform this at a comfortable pace while continuing to fix his gaze on the central gray cursor. At the transition between an EX block and the next VF block, the orange sphere disappeared, and the central gray cursor simultaneously changed from gray back to white. In order to maintain participant attention during the ID blocks, their durations were skewed towards shorter lengths:  80% of the ID durations were 3, 4, or 5 s (3 s x 3, 4 s x 3, 5 s x 2) while 20% were 6 s or 7 s (1 each). The EX blocks varied between 11 s and 15 s such that the combined duration of the EX block and its preceding ID block was 18 s, matching the length

of the VF blocks (18 s). Thus, the length of each VF-ID-EX cycle was fixed at 36 s. The corner ball locations were pseudorandomized but constrained so that back-to-back repetitions were excluded to reduce fatigue and maintain attention. The run was 6.3 min long with a TR of 3 s, starting and ending with VF blocks (11 VF blocks, 10 ID blocks, 10 EX blocks, i.e. 10.5 VF-ID-EX cycles), sampling the brain at 126 time-points (Supplementary Fig. S4C). Two runs were acquired. All task stimuli were presented visually using digital fiber-optic OLED goggles controlled by nordicAktiva stimulus presentation software (NordicNeuroLab, Bergen, Norway). All task stimuli were explained to the participant and practiced outside of the scanner immediately prior to imaging.

The Eye Movement task had a similar hybrid block/event-related paradigm task structure. This task was used to target and, if possible, distinguish the dominant cortical regions engaged during volitional eye movements, also referred to as the frontal eye fields (FEF), and their preparation. The task structure was identical to the object reach with instructed delay task, except that during the EX blocks, the participant was asked to repetitively shift his gaze back and forth between the central gray cursor and the sphere – movements he could physically accomplish. Two runs were acquired.

**fMRI Signal processing: Voxel-based and Surface-based fMRI processing** The fMRI data were analyzed using two parallel processing streams, one voxel-based and the other surface-based, performed independently by different investigators (BRB and NS, respectively). The use of two independent analysis methods leveraged both stream's strengths for data visualization and ensured that the results were robust to differences in analysis methodology (See Fig. S5 – S7).

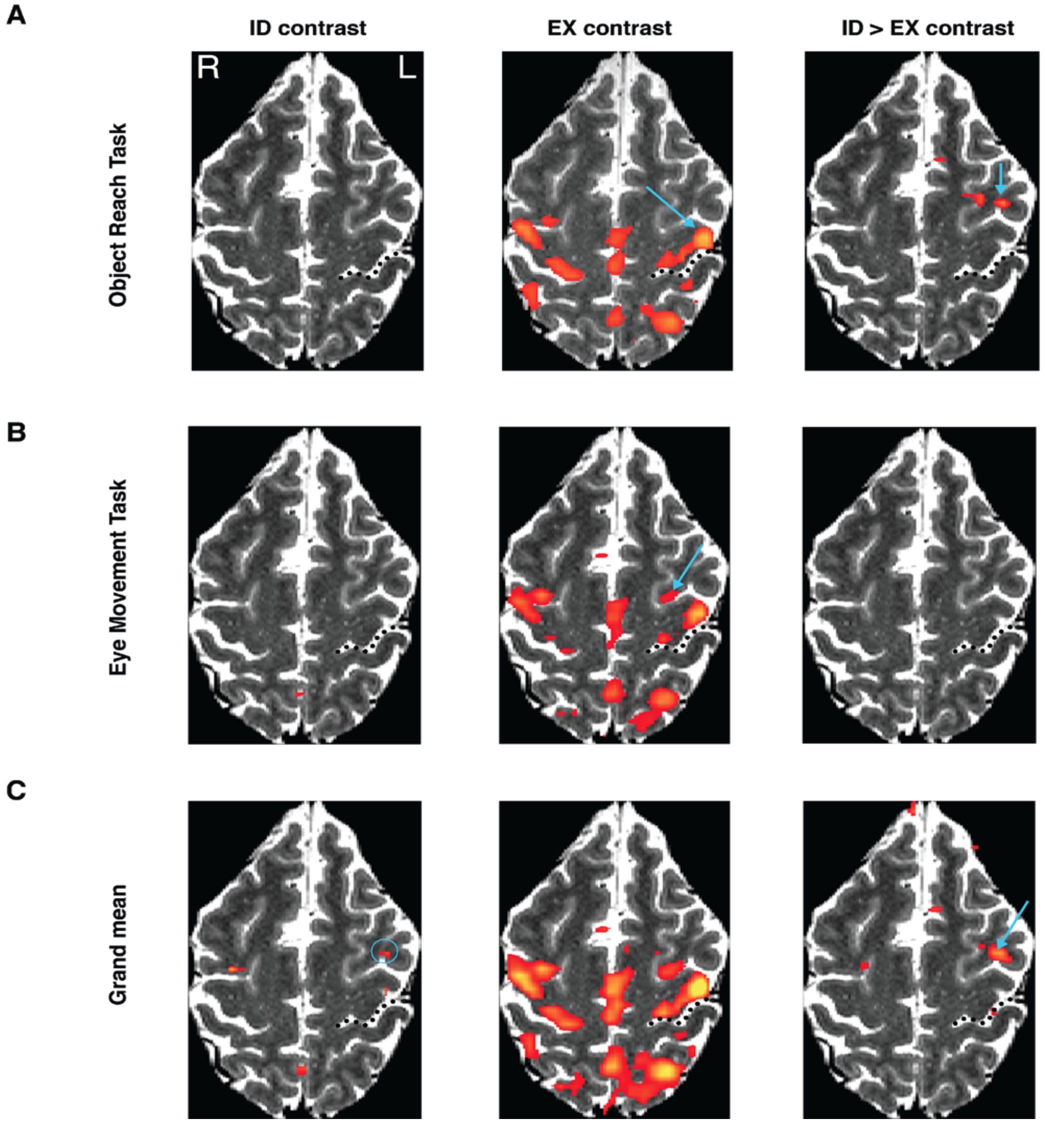


**Fig. S5. Representative statistical maps of fMRI task activation from the voxel-based analysis.**

Object Reach (**A**), Eye Movement (**B**), and grand mean task (**C**) task activations superimposed on axial T2-weighted images at a statistical threshold of $p<0.05$ corrected. **(A)** Object Reach task instructed delay (ID) contrast shows no statistically significant activation, whereas the execution (EX) contrast shows activations in the PCG (*blue arrow*), medial SFG (SMA), post-central gyrus and superior parietal lobule region. The Object Reach task ID > EX contrast brings out focal activation in caudal MFG, anterior to superior precentral sulcus and lateral to superior frontal sulcus; activation region was designated as the target region for upper-limb movement preparation activity (*blue arrow*). **(B)** Eye Movement task, ID contrast, shows no significant activation at target upper-limb movement preparation region, while EX contrast, shows similar activation patterns to the Object Reach EX contrast. The statistically significant activation along banks of the superior precentral sulcus was designated as the FEF region (*blue arrow*). Eye Movement task ID > EX contrast fails to activate target upper-limb preparatory region or other activation regions. **(C)** Combined Object Reach and Eye Movement tasks ID contrast brings out a small focus of activation at target upper-limb preparatory region (*blue circle*), while combined task EX contrast shows stronger but similar activation patterns to that of **(A)** and **(B)**. As expected, the combined task ID > EX contrast brings out a more robust activation focus at target upper-limb preparatory region (*blue arrow*). This figure was compiled using FSL (FSL: FMRIB Software Library 2018) using FEAT (FMRI Expert Analysis Tool) version 6.00, and was labelled using Adobe Illustrator CC 2019.

Voxel-based fMRI processing was performed in FSL (FSL: FMRIB Software Library 2018; Smith 2004) using FEAT (FMRI Expert Analysis Tool) version 6.00. A motion correction algorithm corrected for small head movements (MCFLIRT) [68]. A masking procedure excluded non-brain structures (BET) [69]. Spatial smoothing enhanced signal-to-noise ratio (Gaussian kernel, FWHM 5 mm). High-pass temporal filtering removed low frequency drifts from the BOLD time series (Gaussian-weighted least squares straight line fitting, filter cutoff 90 s, corresponding to sigma = 45 s). After preprocessing, each BOLD run underwent a first-level voxel-by-voxel general linear model (GLM) statistical analysis (FILM) [70]. The main regressors were the predicted BOLD responses for the EX and ID task conditions relative to the VF baseline (Supplementary Fig. S4C). For each condition, the BOLD response model was computed by convolution of its box-car time-course with the canonical FSL double-gamma variate hemodynamic response function. The temporal derivatives of the main regressors were included as nuisance regressors to account for small time shifts between the canonical and actual BOLD responses, as occurs with differences in slice acquisition time. The motion parameters derived from the motion correction algorithm (three for translations and three for rotations) were included as nuisance regressors to account for residual motion-related variance in the BOLD signal. Local pre-whitening was used to account for temporal autocorrelations in the BOLD time series.

The GLM analysis produced statistical maps of task activation (Supplementary Fig. S5) for the contrasts of interest: the EX condition relative to the VF baseline (the EX contrast), the ID condition relative to the VF baseline (the ID contrast), and the ID condition relative to the EX condition, (the ID > EX contrast). Specifically, if $\beta_{\mathrm{EX}}$ and $\beta_{\mathrm{ID}}$ represent the GLM coefficients for the BOLD hemodynamic response regressors corresponding to the EX and ID conditions relative to the VF baseline, then the contrast vectors corresponding to the EX, ID, and ID > EX contrasts are [1, 0], [0, 1], and [-1, 1], respectively (ignoring the nuisance regressors in the contrast vectors, which are all 0). These contrasts test the three null hypotheses $H_0\colon \beta_{\mathrm{EX}} = 0$ , $H_0'\colon \beta_{\mathrm{ID}} = 0$ , and $H_0''\colon \beta_{\mathrm{ID}} - \beta_{\mathrm{EX}} > 0$ , using one-sided *t*-tests. For task paradigms that were performed twice, second level statistical analyses were performed to produce maps of mean activation using a fixed effects

model. To further focus on activation related to the instructed delay condition, the four runs incorporating an instructed delay (two Object Reach, two Eye Movement) were combined in a second level fixed effects analysis to produce maps of mean activation for contrasts ID and ID > EX (Fig. S4A, B). Voxel-based *Z* (Gaussianized *T*) statistics were controlled for family-wise error rate (FWER) using Gaussian random field theory at a significance level of $\alpha < 0.05$, subsequently denoted by $p<0.05$, corrected [71]. Statistical maps were coregistered to high resolution T1- and T2-weighted images using FLIRT [68,72].

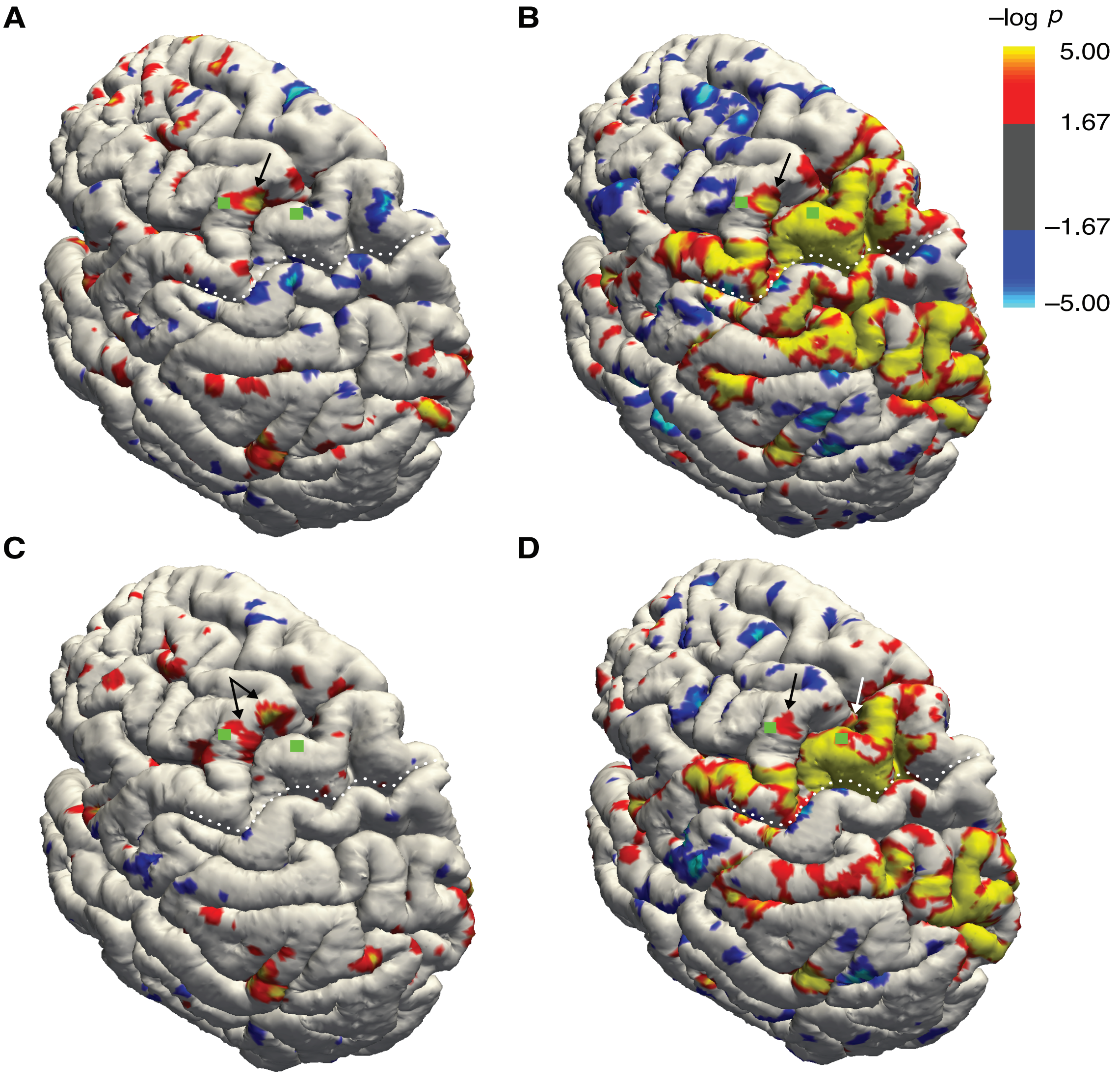


**Fig. S6. Statistical maps of fMRI task activation in the left cerebral hemisphere from the surface-based analysis.**

Positive and negative activations, superimposed on the pial surface at a one-sided statistical threshold of $p<0.01$, uncorrected. The surface- and voxel-based activations were largely concordant, noting slightly different

statistical methodologies and thresholds. (**A**) The Object Reach task, ID contrast, elicited largely isolated activation in the middle frontal gyrus (*black arrow*), which was subthreshold for this contrast on the voxel-based analysis. **(B)** As in the voxel-based analysis, the Object Reach task, EX contrast, showed significant activation in PCG, SMA, S1, SPL/IPS, and candidate FEF. The candidate FEF activation is not fully visualized from the surface, because it is mostly enfolded along the banks of the superior precentral sulcus. Significant activation was also seen at the target upper-limb preparatory region (*black arrow*), which was not seen for this contrast in the voxel-based analysis. **(C)** The Eye Movement task, ID contrast, activated two different MFG regions, possibly representing an additional site involved in movement preparation (*black arrows*). **(D)** As in the voxel-based analysis, the Eye Movement task, EX contrast, elicited similar activations in a distribution surprisingly similar to the Object Reach task, including FEF (*white arrow*), but also M1, SMA, SPL/IPS, and near the implanted upper-limb preparatory region (*black arrow*), while largely excluding S1. Color scale (**B**) is similar across A-D. This figure was generated using FreeSurfer v6.0 and FS-FAST (Fs-FAST: FreeSurfer Functional Analysis Stream 2011), and was labelled using Adobe Illustrator CC 2019.

Volume renderings of the coregistered structural images and statistical maps were generated using MRIcro v. 1.9.0 (MRIcro c2012) (Supplementary Figure S6). Surface-based fMRI processing was performed using FreeSurfer v. 6.0 [73] and FS-FAST (FsFast: FreeSurfer Functional Analysis Stream 2011). In contrast to voxel-based strategies, FreeSurfer constructs 2D models of the cortical pial and grey-white boundaries, accurately delineating gyral folds and sulcal furrows. fMRI analyses using FsFast exploit this surface-based strategy, limiting spatial smoothing to grey matter voxels defined by the cortical surface models, and reducing the effect of smoothing across cortical folds that may otherwise decrease statistical power. Anatomical processing steps included intensity corrections of the T1-MEPRAGE images, segmentation of grey matter and white matter voxels, creation of a 2D model of the outer white matter surface, and the creation of an outer grey matter surface model, which was driven by algorithmic smoothness constraints and intensity information from the T1-MEMPRAGE and SPACE-T2-FLAIR images. For each BOLD run, FS-FAST fMRI analysis included motion correction, slice timing correction, registration to the anatomical images (FreeSurfer bbregister) [74], and spatial smoothing over the cortical surface (FWHM 2 mm). Surface-based statistical parametric maps were generated for the EX and ID contrasts using a GLM, incorporating double-gamma variate modeling of the EX and ID hemodynamic BOLD responses, second order polynomial modeling of low-frequency drifts, and pre-whitening to account for temporal autocorrelations. The two runs each of the object reach and eye movement tasks were combined to generate statistical maps of mean activation for the EX and ID contrasts

(Fig. S6). In each case, the surface-based statistical threshold was set at $p<0.01$ without correction for multiple comparisons.

**Final selection of array locations** Despite the somewhat different methodologies, the voxel-based and surface-based mapping yielded closely corresponding anatomical locations for key upper-limb movement preparation and execution activation foci (Supplementary Fig. S7).

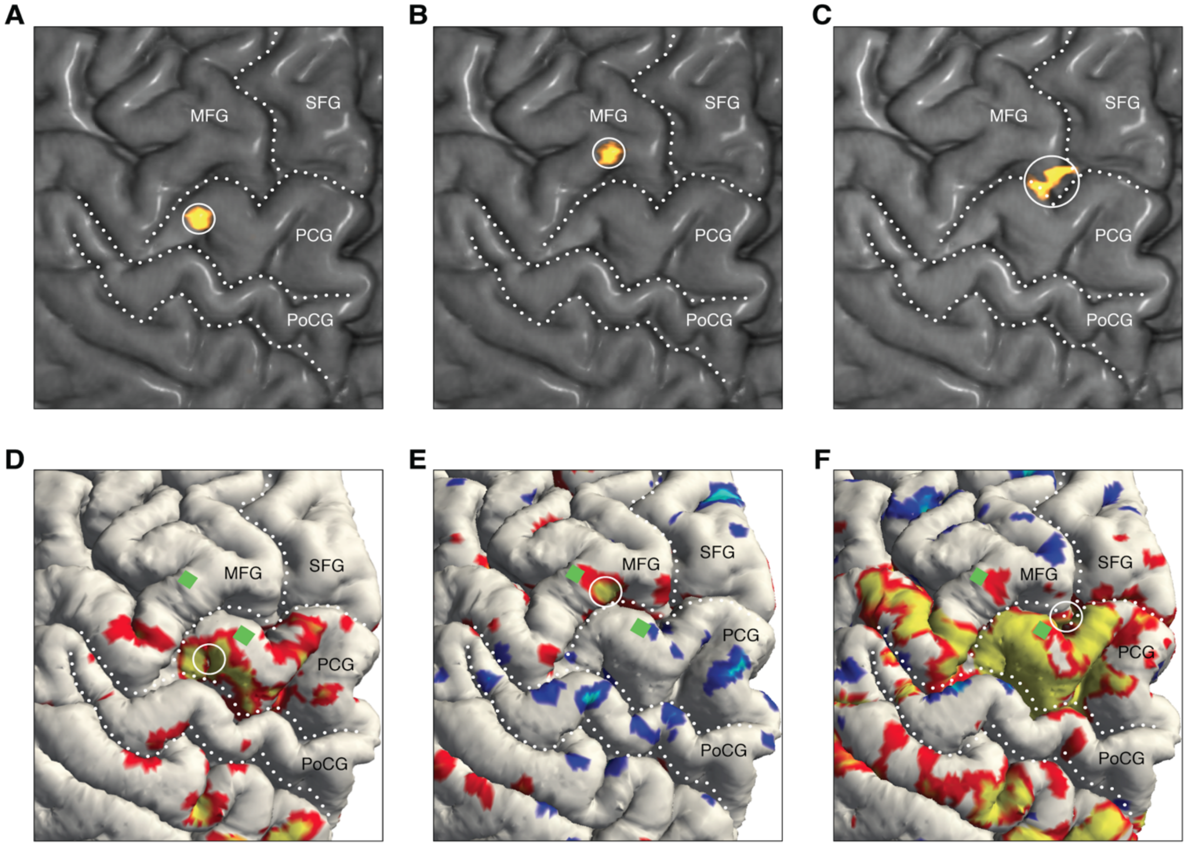


**Fig. S7. Surface mapping of upper-limb movement execution, movement preparation, and FEF activation foci.**

Surface mapping of independent voxel-based **(A-C)** and surface-based **(D-F)** functional analyses. In the voxel-based analysis, the activation sites are projected approximately orthogonally to the pial surface. In the surface-based analysis, they are intrinsically constrained to the pial surface. Circles enclose the peak activation sites. Dotted lines highlight the superior frontal, superior precentral, central, and postcentral sulci. In (**D-F)** green squares indicate the sites (as determined by CT-MR fusion) of the surgically-placed microelectrode array implants targeting the upper-limb movement execution activity (PCG) and the movement preparation activity (MFG). The three key activation foci in the targeted upper-limb movement execution, movement preparation, and the FEF regions were highly concordant between the two independent analyses, noting slightly more anterior positions on the voxel-based analysis, likely reflecting slight differences in structural-functional image coregistration. Activation sites were derived from the following contrasts and visualized at the following statistical thresholds: **(A)** Object Reach task, mean EX contrast, displayed at a threshold of Z > 10.0, where Z > 5.0 corresponds to p<0.05, corrected. **(B)** Combined Object Reach and Eye Movement tasks, ID > EX contrast, displayed at a threshold of Z > 3.2, where Z > 2.3 corresponds to p<0.05, corrected; masked to isolate target

MFG. **(C)** Eye Movement task, EX contrast, displayed at a threshold of Z > 6.0, where Z > 5.0 corresponds to p<0.05, corrected; masked to isolate FEF activation. **(D)** Object Reach, EX contrast, displayed at a threshold of p<0.01, uncorrected. **(E)** Object Reach, ID contrast, displayed at a threshold of p<0.01, uncorrected. **(F)** Eye Movement, EX contrast, displayed at a threshold of p<0.01 uncorrected. (SFG, superior frontal gyrus; MFG, middle frontal gyrus; PCG, precentral gyrus; PoCG, postcentral gyrus.). Images (**A – C**) were created using MRIcro v. 1.9.0 (MRIcro c. 2012) and FSL: (FMRIB Software Library 2018; Smith 2004) using FEAT (FMRI Expert Analysis Tool) version 6.00, images **(D - F)** were generated by FreeSurfer v6.0 and FS-FAST (Fs-FAST: FreeSurfer Functional Analysis Stream 2011); labeling was done with Adobe Illustrator CC 2019.

Activation in the target upper-limb movement execution region was localized to the crown of the PCG, in the ventrolateral aspect of the PCG "hand knob" area (Supplementary Fig. S7A, D). Activation in the target upper-limb movement preparation region is localized to the crown of the MFG, anterior and slightly dorsomedial to the site of target movement execution region in PCG (Supplementary Fig. S7B, E). Activation in the designated FEF region was localized to the banks of the superior precentral sulcus, just ventrolateral to its junction with the superior frontal sulcus (Supplementary Fig. S7C, F). During the surgical procedure, the final location of the array targeting the upper-limb movement execution was selected to be as close as possible to the corresponding peak activation site while ensuring that it was placed in a mechanically stable position near the crown of the gyrus, devoid of cortical veins (Fig. 1 and Fig. S7D). The final location of the array targeting upper-limb movement preparation was selected to be as close as possible to the corresponding peak activation site, given the same surgical constraints and away from the FEF region (Fig. 1 and Fig. S7E).

While the human PCG is commonly referred to as either "primary motor cortex" or "motor cortex" in clinical and clinical research communities, we note here that this anatomic-functional assignment may not be fully accurate [75,76]. Most historical and recent work with single neuron recordings in non-human primate primary motor cortex has referred to recordings derived from the anterior bank of the central sulcus, where there is general agreement that there is a substantial population of large Betz cells which histologically define Brodmann area 4 [77]. In humans, however, these large Betz cells taper in density as the surface of the precentral gyrus is approached[46,78]. It is not clear if recordings performed at the crown of the precentral gyrus — where single neurons are being recorded by the planar array of microelectrodes placed by current

human clinical research groups — should be considered, at least on anatomic criteria alone, as being seated in the primary motor cortex.

**Electrophysiology signal acquisition during iBCI research session** Electrophysiology recordings were carried out during three research sessions on post-implant trial days 356, 357, and 362. At the start of each research session, microelectrode array signals were inspected visually using the Cerebus Central Spike Panel (Blackrock Microsystems) to identify channels whose signals appeared overly contaminated by environmental electromagnetic noise. These electrodes were then excluded from consideration during feature selection for real-time decoding. Electrodes where the mean rate of threshold crossings fell below 1 Hz or exceeded 1 kHz were also excluded. Each research session began with a one-minute reference block during which the participant was not engaged in a task. The raw recordings from this block were used to determine a subset of 40 channels per array to be used for Common Average Referencing (CAR) [79], specifically those with the lowest root-mean-square values. The reference block was also used to compute the spike threshold per channel, set at -3.5 times the channel's standard deviation. In all subsequent blocks of a research session, the real-time computer first down sampled the 30 kHz data to 15 kHz, before applying CAR online. Using 20ms bins with a 4ms buffer to reduce edge effects, we then non-causally filtered for action potentials between 250 - 5000 Hz with an 8th-order Butterworth filter [80]. The power in this band constitutes the first neural feature used for our online decoding; the rate of threshold crossings constitutes the second [81,82]. Each of these features was normalized using the means and standard deviations calculated in the previous block.

**iBCI decoder calibration** For calibration, a center-out task with eight radially distributed circular targets was presented on an LCD monitor placed 55-60 cm away from the participant, using custom MATLAB software (MathWorks, Natick, MA). Peripheral targets were cued in a pseudo-random order and interleaved with center target cues. The participant controlled the BCI using his preferred imagery: movement of his right pointer finger and holding in the direction of the target. A trial was considered successful if, within a timeout period of five seconds, the participant placed the

cursor in contact with the cued target for 1 second. Following an unsuccessful trial, the cursor would move automatically to the intended target, and the next trial was cued.

At the start of the calibration block, the parameters of a real-time steady-state Kalman filter [83-85]were initialized to values that leave the cursor stationary. These parameters were then updated every 2-5 seconds by custom software running parallel to the real-time model [86], using inferred movement intention (unit vectors pointing from the cursor to the cued target) as labels for the observed neural features. Any decoded velocity whose magnitude exceeded a set threshold (the 66th percentile of all magnitudes, learned from the latest calibration block) was used to iteratively estimate an undesired bias in BCI control (see [4] for details). This estimate was used to adjust the decoded velocity before being sent to the cursor.

**Table S1: Number of neural features from each array used to control iBCI cursor across sessions.**

| | Eye-Hand iBCI Task | | Multi-modal iBCI Task | |
|---|---|---|---|---|
| | MFG (power/rate) | PCG (power/rate) | MFG (power/rate) | PCG (power/rate) |
| **Session 1** | 0/0 | 18/2 | 3/1 | 38/26 |
| **Session 2** | 5/1 | 26/6 | 4/1 | 37/19 |
| **Session 3** | 9/2 | 21/9 | 7/1 | 39/20 |

Based on previous sessions with good neural control, the top 20 features were selected according to their SNR ranking [87] for the Eye-Hand task. For the Multi-modal Cues task, the number of features was increased to 40. The change in the number of features did not affect the quality of cursor control or impact our reported findings, as the data from the Multi-modal Cues task was analyzed separately from the Eye-Hand task data. The calibration block ended after four minutes, and the filter parameters at the end of the block were carried forward to the task blocks. Filters were updated from the movement epochs of the task if control was deemed poor. A summary of unique features used from each array across both tasks is shown in Supplemental Table S1. Due to multiple filter builds, total unique features vary from the 20, 40 features used in the Eye-Hand, Multi-modal Cues task, respectively.

**Trial rejection** The three criteria for trial rejection were *unsuccessful trials*, *insufficient fixation*, and *false starts*. Here, we describe the trial exclusion criteria for both attempted hand movements and eye movements (Eye-Hand task used in the preliminary analysis; see Supplementary text) *Unsuccessful trials:* Trials in which the participant selected the incorrect target or was unable to select a target within the timeout period. *Insufficient fixation:* For the eye-control condition of the Eye-Hand task, these were trials in which the participant's eyes moved away from the center circle, beyond the fixation threshold used during the task. For the hand-control condition of the Eye-Hand task, these were trials in which the hand-cursor moved away from the center circle, beyond the hand fixation threshold used during the task. *False starts:* Hand-control trials in which the participant appeared to move the cursor towards the cued target prematurely. The threshold used to detect false starts was determined empirically, as follows: i) isolate the baseline phases for all trials in the given task, ii) compute the hand cursor's displacement within non-overlapping windows of 100ms throughout the baseline phase, iii) use the $99^{th}$ percentile of the resulting distribution as the threshold. Any trials in which the hand cursor position exceeded this threshold 100ms after the go cue were considered false starts [88]. Based on these criteria, the total number of trials was 354 for the eye control condition, 320 for the hand control condition of the Eye-Hand task, and 872 for the Multi-modal Cues task. Note that trial rejection was not applicable to the Passive Listening task.
